# Cryo‐electron microscopy structure of CLHM1 ion channel from *Caenorhabditis elegans*

**DOI:** 10.1002/pro.3904

**Published:** 2020-06-30

**Authors:** Weixin Yang, Youwang Wang, Jianli Guo, Lingli He, Ye Zhou, Hui Zheng, Zhenfeng Liu, Ping Zhu, Xuejun C. Zhang

**Affiliations:** ^1^ National Laboratory of Biomacromolecules, CAS Center for Excellence in Biomacromolecules Institute of Biophysics, Chinese Academy of Sciences Beijing China; ^2^ College of Life Sciences University of Chinese Academy of Sciences Beijing China

**Keywords:** CLHM1, cryo‐EM, elegans, structure

## Abstract

Calcium homeostasis modulators (CALHMs/CLHMs) comprise a family of pore‐forming protein complexes assembling into voltage‐gated, Ca^2+^‐sensitive, nonselective channels. These complexes contain an ion‐conduction pore sufficiently wide to permit the passing of ATP molecules serving as neurotransmitters. While their function and structure information is accumulating, the precise mechanisms of these channel complexes remain to be full understood. Here, we present the structure of the *Caenorhabditis elegans* CLHM1 channel in its open state solved through single‐particle cryo‐electron microscopy at 3.7‐Å resolution. The transmembrane region of the channel structure of the dominant class shows an assembly of 10‐fold rotational symmetry in one layer, and its cytoplasmic region is involved in additional twofold symmetrical packing in a tail‐to‐tail manner. Furthermore, we identified a series of amino acid residues critical for the regulation of *Ce*CLHM1 channel using functional assays, electrophysiological analyses as well as structural‐based analysis. Our structure and function analyses provide new insights into the mechanisms of CALHM channels.

## 1 INTRODUCTION

Calcium homeostasis modulator 1 (CALHM1) was originally identified in the human hippocampus^1^ and was postulated to be related to Alzheimer's disease. Recently, a large number of genes homologous to *calhm1* encoding a pore‐forming protein have been identified across many species,^2^ and shown to be involved in the regulation of diverse biological functions. For instance, six human CALHM homologs (CALHM1–6) have been identified in a wide range of tissues.^2^, ^3^ One member, CALHM1 has been shown to participate in taste signaling.^4^, ^5^ In the plasma membrane, CALHMs form glycosylated homomeric^3^ (or heteromeric^6^) ion channels that are voltage‐gated, Ca^2+^‐sensitive, and nonselective. Each channel contains a large pore permeable to a diverse range of ions, including Ca^2+^, K^+^, Cl^−^, and ATP, as well as to a variety of fluorescent dyes.^4^, ^7^, ^8^, ^9^ Intriguingly, a previous study in mouse indicated that ATP molecules function as a neurotransmitter diffusing from taste bud cells through the CALHM1 channels to excite downstream nerve cells.^9^ In particular, these channels are located in patches near the intercellular interface, adjacent to specialized large mitochondria. Recently, the structures of human CALHM2 (hCALHM2), hCALHM4, hCALHM6, and CALHM1 homologs from chicken (*Gallus gallus domesticus*), killifish (*Oryzias latipes*), and *Caenorhabditis elegans* have been reported^10^, ^11^, ^12^, ^13^ (Table S1). For instance, the oligomers of hCALHM2 subunits form a hemichannel that exhibits an 11‐fold rotational symmetry in its transmembrane (TM) region. In addition, two such undecameric hemichannels form a double‐layered gap‐junction type structure with their extracellular sides being packed in a head‐to‐head manner.

*C. elegans* expresses only a single homolog of CALHM1, termed *Ce*CLHM1, in its sensory neurons and muscle cells.^14^ In these cells, *Ce*CLHM1 forms a Ca^2+^‐sensitive ion channel involved in regulating the function of excitable cells and maintaining normal motility of the worms. *Ce*CLHM1 shares similar channel properties with human CALHM1, such as being inhibited by the nonselective blockers Ruthenium Red (RuR)^15^ and Gd^3+^, as well as by increasing the extracellular Ca^2+^ concentration ([Ca^2+^]_o_).^2^, ^3^, ^14^ Despite strong functional conservation, the *Ce*CALHM1 protein shows significant differences in the primary sequence compared with those from human and mouse (e.g., 17% sequence identity and 33% sequence similarity to hCALHM1, and 22% sequence identity and 31% sequence similarity to hCALHM2) (Figure S1). In addition, the pharmacological properties of *Ce*CLHM1 are distinct from those of other types of known gap junction channels, such as connexins, innexins, and pannexins, which were proposed to share similar pore‐lining structures with CALHM channels.^16^ For instance, blockers of connexin and pannexin, namely, octanol and carbenoxolone, cause a nearly twofold increase (instead of decrease) of ion permeability in *Ce*CLHM1.^14^ In the current study, we report the cryo‐electron microscopy (cryo‐EM) structure of the *Ce*CLHM1 channel, and show that two homodecameric hemichannels are packed with each other along the pore axis in a tail‐to‐tail mode distinct from most known structures of CALHMs, including a reported *Ce*CLHM1 structure,^11^ as well as other types of gap junctions.^17^, ^18^, ^19^ Mechanisms of channel regulation are proposed based on conserved structural features of the CALHM family.

## 2 RESULTS

### 2.1 *Overall structure*

**FIGURE 1:**
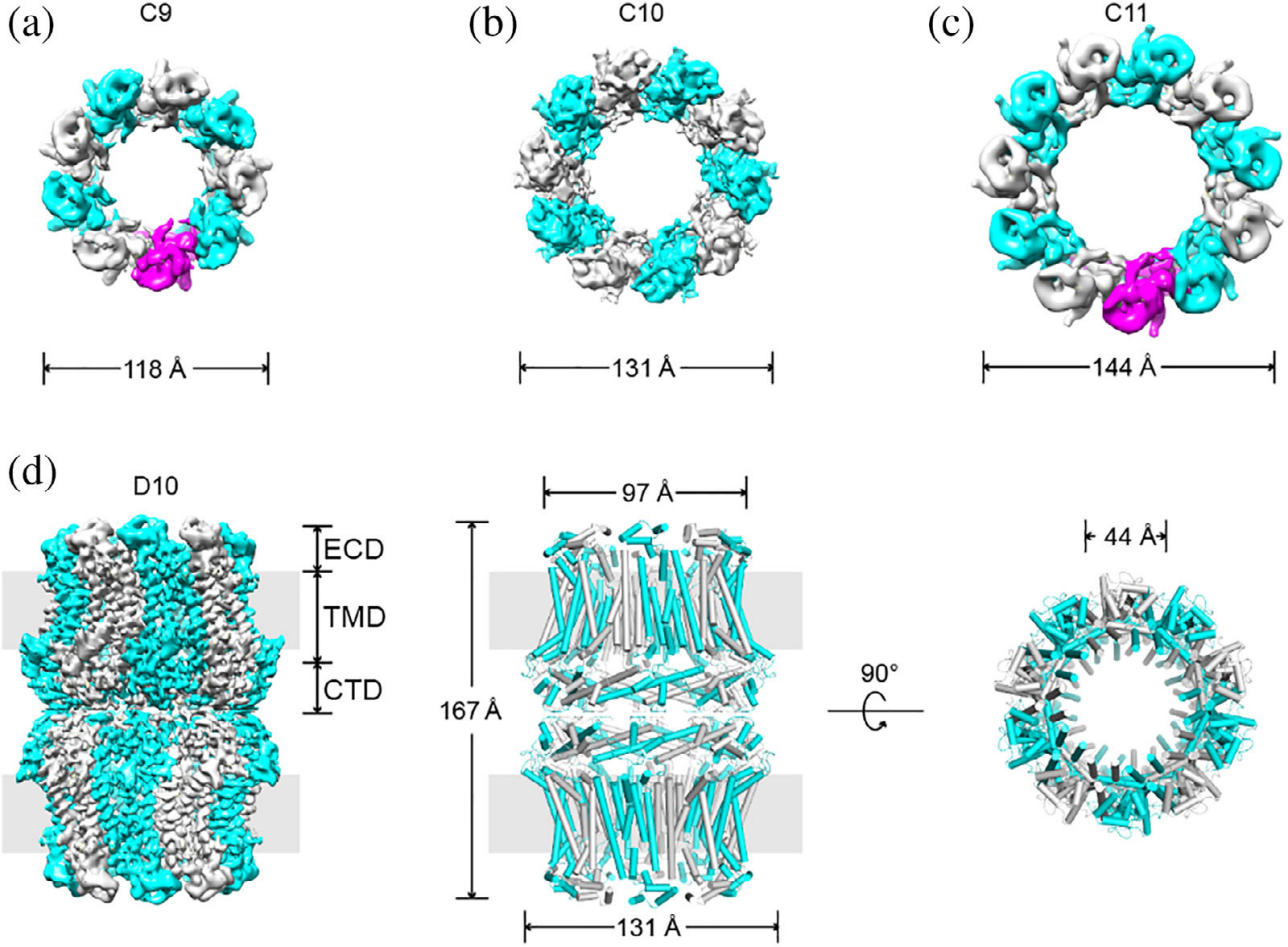
Architecture of the CLHM1 channel. (a–c) Cryo‐electron microscopy (cryo‐EM) density map of the *Ce*CLHM1 (hemi‐)channel in the absence of BPY, displaying nonamer, decamer, and undecamer forms. Protomers are colored alternatingly in cyan and gray, and additionally in magenta for both nonamer and undecamer forms. (d) Cryo‐EM density map of *Ce*CLHM1‐BPY complex at 3.73 Å resolution (left) and atomic model of *Ce*CLHM1 displaying the 20 subunits, in a side view (middle) and top view (right). CTD, C‐terminal domain; ECD, extracellular domain; TMD, transmembrane domain

**FIGURE 2:**
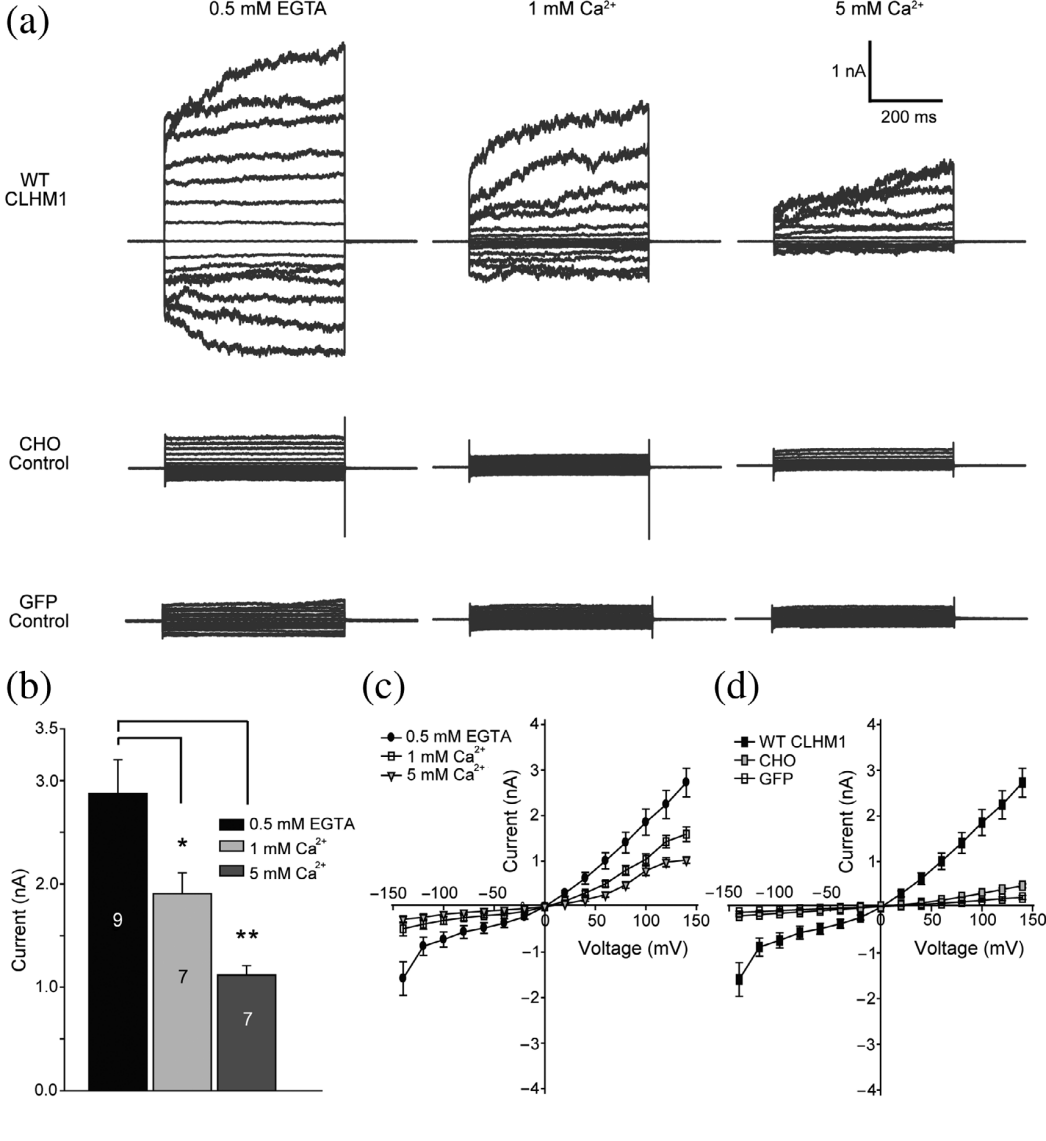
Whole‐cell patch clamp recording of the channel activities of *Ce*CLHM1 proteins expressed in CHO cells. (a) Representative current–voltage relationships were obtained by applying 500 ms voltage pulses ranging from +140 to −140 mV (in 20 mV steps) to cells expressing wild‐type (WT) *Ce*CLHM1, CHO control cells, and CHO cells transfected with green fluorescent protein (GFP)‐alone vector. Currents were recorded in the presence of 0.5 mM ethylene glycol bis(2‐aminoethyl)tetraacetic acid (EGTA) (0 mM Ca^2+^), 1 mM Ca^2+^, and 5 mM Ca^2+^. The holding potential was set at 0 mV before the voltage pulses were applied. (b) Statistics on the average values of the currents recorded at +140 mV in the presence of 0.5 mM EGTA, 1 mM Ca^2+^ or 5 mM Ca^2+^ (*, *p* < .05; **, *p* < .01, Student's *t* test). Number of measurements (*n*) is labeled on the bar; error bars indicate *SEM*. (c) The current–voltage relationship plots of WT *Ce*CLHM1 data as represented in (a). Data are presented as mean ± *SEM* (*n* ≥ 7). (d) Averaged current amplitudes measured under different voltage settings with cells expressing WT *Ce*CLHM1 (*n* = 9), CHO control cells (*n* = 7), and cells transfected with GFP‐alone vector (*n* = 8) in the presence of 0.5 mM EGTA. Data are plotted as mean ± *SEM*

To understand the structural bases of CALHM mechanisms, we performed structure analysis on *Ce*CLHM1. The *C. elegans clhm‐1* gene was expressed in HEK293F cells. The recombinant full‐length *Ce*CLHM1 protein was purified to homogeneity with the detergent lauryl maltose neopentyl glycol (LMNG), either containing or lacking an enhanced green fluorescent protein (GFP) fused to the C‐terminus (Figure S2). The structure of full‐length *Ce*CLHM1 channel was solved using the single‐particle cryo‐EM method (Figures S4 and S5). The hemichannels formed by *Ce*CLHM1 showed multiple oligomeric forms, namely nonamers (35%), decamers (58%), and undecamers (7%). As shown in Figure 1a–c, all forms exhibited their axis of rotational symmetry as being perpendicular to the membrane plane. In the absence of the C‐terminal GFP tags, these membrane‐spanning hemichannels further assembled with each other into double‐layered dimers. Among these dimers, the double‐decameric form dominated. The particles were reselected with the D10 symmetry being imposed, and the structure of the *Ce*CLHM1 channel was determined in the 20‐mer complex form (Figure 1d). The *Ce*CLHM1 protein was also transiently expressed in CHO cells, and electrophysiological analysis of these cells confirmed that the recombinant protein channel maintained its voltage‐ and Ca^2+^‐sensitive properties (Figure 2), consistent with previous reports.^14^

While the RuR molecule binds to the hCALHM2 channel, traps the channel in an inhibited state,^13^ and also inhibits *Ce*CLHM1,^14^ the addition of RuR to the *Ce*CLHM1 sample caused severe aggregation during preparation of the cryo‐EM sample. The cryo‐EM data collected from the sample with RuR were of low quality and not further processed. Through a parallel effort in solving the structure of *Ce*CLHM1, we found that addition of BPY ((2,2′‐bipyridyl)‐dichlororuthenium(II), a ruthenium‐containing compound (Figure S2d) to the sample assisted in forming the double‐layered channel, despite that no densities accounting for the BPY molecules were found. The existence of nonamers, decamers, undecamers as well as their dimerization was BPY independent, although addition of BPY enhanced the population of the dimer of decamers and significantly reduced orientation preference. These effects of BPY suggest that it may either bind at the interface between the two hemichannels or induce a conformational change of the C‐terminal region at the packing interface to facilitate the observed symmetrical dimerization.

**FIGURE 3:**
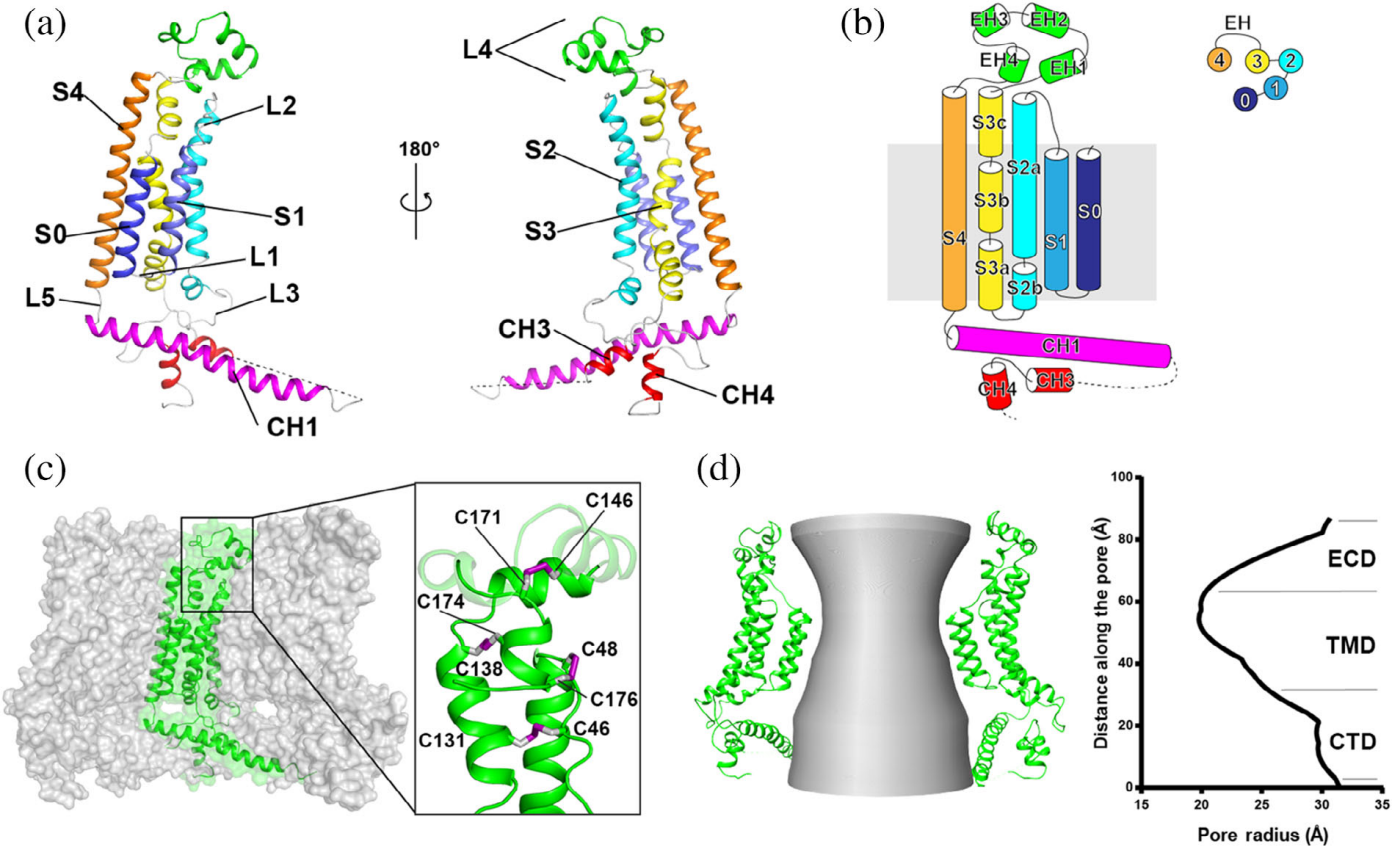
A protomer of the *Ce*CLHM1 channel and the pore size. (a,b) Atomic model of a *Ce*CLHM1 protomer in ribbon representation. Helices S0–S4, Loops L1–L5, and C‐terminal domain (CTD) composed of CH1–CH4 are labeled. (c) Illustration of the protomer of *Ce*CLHM1 within the overall channel structure. The insert is a magnified view of the intramolecular interface between extracellular Loop‐2 and Loop‐4. The four disulfide bonds are colored purple. (d) Channel inner surfaces of *Ce*CLHM1 and radius along the pore axis measured with the HOLE program^39^

Based on the cryo‐EM density map, we built a model of the *Ce*CLHM1 channel de novo, and refined it at an overall resolution of 3.73 Å. As shown in Figure 1, the double‐layered 20‐mer channel spans ~170 Å in length along the direction of the membrane normal, and is ~130 Å in width. The channel contains a wide funnel‐like pore with a diameter of approximately 40 Å around the narrowest central region, and over 60 Å around the extracellular and intracellular entrance (Figure 3). Thus, the structural model of *Ce*CLHM1 represents an open‐state form of the channel, as any other structural adjustment would probably further constrict the pore size. In contrast to the head‐to‐head packing observed in most other reported structures of CALHM,^10^, ^11^, ^12^, ^13^ connexins,^19^, ^20^ and innexins,^17^ the doubled‐layered channel of *Ce*CLHM1 is formed by docking the C‐terminal domains (CTDs) from two decameric hemichannels in a tail‐to‐tail manner. In addition, less than 3% particles showed asymmetrical dockings of presumably different hemichannels and were not processed further. In our final model, each protomer subunit contains 294 out of a total of 329 residues. The unobserved parts of *Ce*CLHM1 include Residues 261–278 and 17 C‐terminal residues both in CTD because traceable density were lacking. In addition, the N‐terminal helix (S0) was built as a poly‐Ala model due to lack of side‐chain features in the cryo‐EM map. The refined double‐layered model from the BPY‐containing *Ce*CLHM1 sample showed good stereochemistry and fitted well with the experimental density map (Figures S5 and S6). Statistics of data collection and refinement are summarized in Table S2.

### 2.2 *The protomer structure*

**FIGURE 4:**
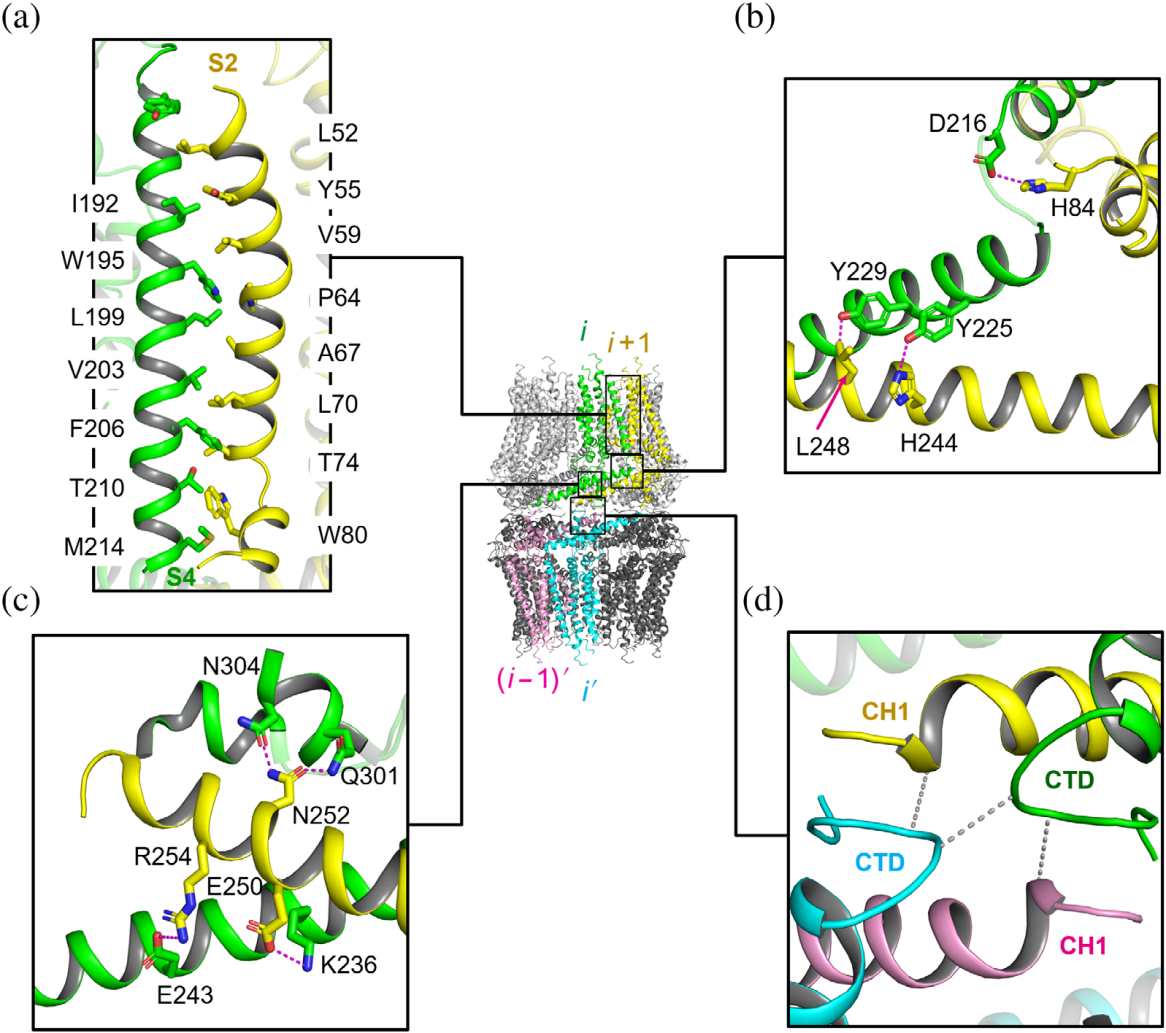
Interactions between subunits of *Ce*CLHM1. (a) Interactions between transmembrane (TM) regions of neighboring subunits. The intersubunit packing is dominated by hydrophobic interactions between S4 of the *i*th subunit and S2 of the (*i* + 1)th subunit. (b) Interactions between C‐terminal domains (CTDs)s of neighboring subunits from the same hemichannel on the cytoplasmic side. Hydrogen bonds are shown in magenta. (c) Second set of interactions between CTDs of neighboring subunits. (d) Docking interactions between the two hemichannels. Four subunits were packed together through a number of electrostatic and hydrophobic interactions

Each of the 20 identical protomer subunits shows an L‐shaped structure (Figure 3a,b). A bundle of five TM helices and a long cytoplasmic helix form the vertical and horizontal arms, respectively. The protomer structure of *Ce*CLHM1 superimposes well with those from reported CALHM channel structures (Table S1). For instance, the root‐mean‐square deviation (RMSD) from hCALHM2 is 1.3 Å. The N‐terminus of *Ce*CLHM1 points to the extracellular side, while the C‐terminus is located on the cytoplasmic side. To be consistent with the nomenclature of the previously reported hCALHM2 structure, we denoted the TM helices as S0–S4. As shown in Figure S6, the density for the *Ce*CLHM1 polypeptide was traceable to the N‐terminal end, although the local resolution was significantly lower than that for the other parts of the structure. S2–S4 helices span the membrane and form the outer wall of the channel (Figure 4a), whereas both S0 and S1 line the interior of the channel and are about one to three helix‐turns shorter at both ends (Figure S6d). S0 and S1 are tightly packed against the outer wall, shaping a widely open pore at the central region of the channel, and the lumen surface of the pore exhibits alternating layers of positive and negative charges (Figure S7). Presumably, it may require dissociation of S0 and S1 from the outer wall and further projection of the N‐terminal region into the pore center for the channel to assume a closed state. In addition, both S2 and S3 are discontinuous, breaking at residues P64^S2^, V75^S2^, P115^S3^, and D125^S3^ (Figure 3c).

On the extracellular side, a long loop (Loop‐4, Residues 137–181) connects S3 to S4. Together with Loop‐2 (Residues 41–49), it forms an extracellular domain (ECD) containing four short helices as well as four intrasubunit disulfide bonds (C46–C131, C48–C176, C138–C174, and C146–C171) (Figure 3d). Loop‐4 is five residues longer than and clearly distinct from its counterpart in hCALHM2, which contains only two disulfide bonds.^13^

The C‐terminal region of *Ce*CLHM1 forms the cytoplasmic CTD. It contains a long helix CH1 and two accompanying short helices, CH3 and CH4 (Figure 3a), following the hCALHM2 nomenclature. The CH2 helix and its connecting loops observed in hCALHM2 are absent in our model of *Ce*CLHM1, probably due to high flexibility of these regions. CH1, CH3, and CH4 are all involved in the interlayer docking site (Figure 4). The interlayer interaction buries approximately 3,800 Å^2^ of solvent‐accessible surface (SAS) area from the two hemichannels, and a significant portion of the interface is contributed by hydrophobic amino acid residues (Figure S7). Fusion of GFP to the C‐terminus of *Ce*CLHM1 prevented formation of the double‐layered channel but did not disrupt formation of the hemichannel (Figure S3), confirming that the C‐terminal region plays a crucial role in mediating the interactions between the two hemichannels. A similar tail‐to‐tail packing has been reported recently for double‐layered decameric/undecameric channels of hCALHM4 (Reference 10), in which detailed information on interlayers interactions are lacked because of structural flexibility. Structure supposition shows that the RMSD between the decameric hemichannels of *Ce*CLHM1 and hCALHM4 is approximately 2.1 Å for ~1,730 pairs of C_α_ atoms (using a 4 Å cutoff), and large deviations are observed in regions of S0 helices, ECDs, and S3a helices. Furthermore, once a pair of hemichannels is supposed with each other, the other pair of hemichannels differs by a rotation of approximately 13° and 10 Å shift along the center axis.

### 2.3 *Structure‐based mutagenesis and functional analysis*

**FIGURE 5:**
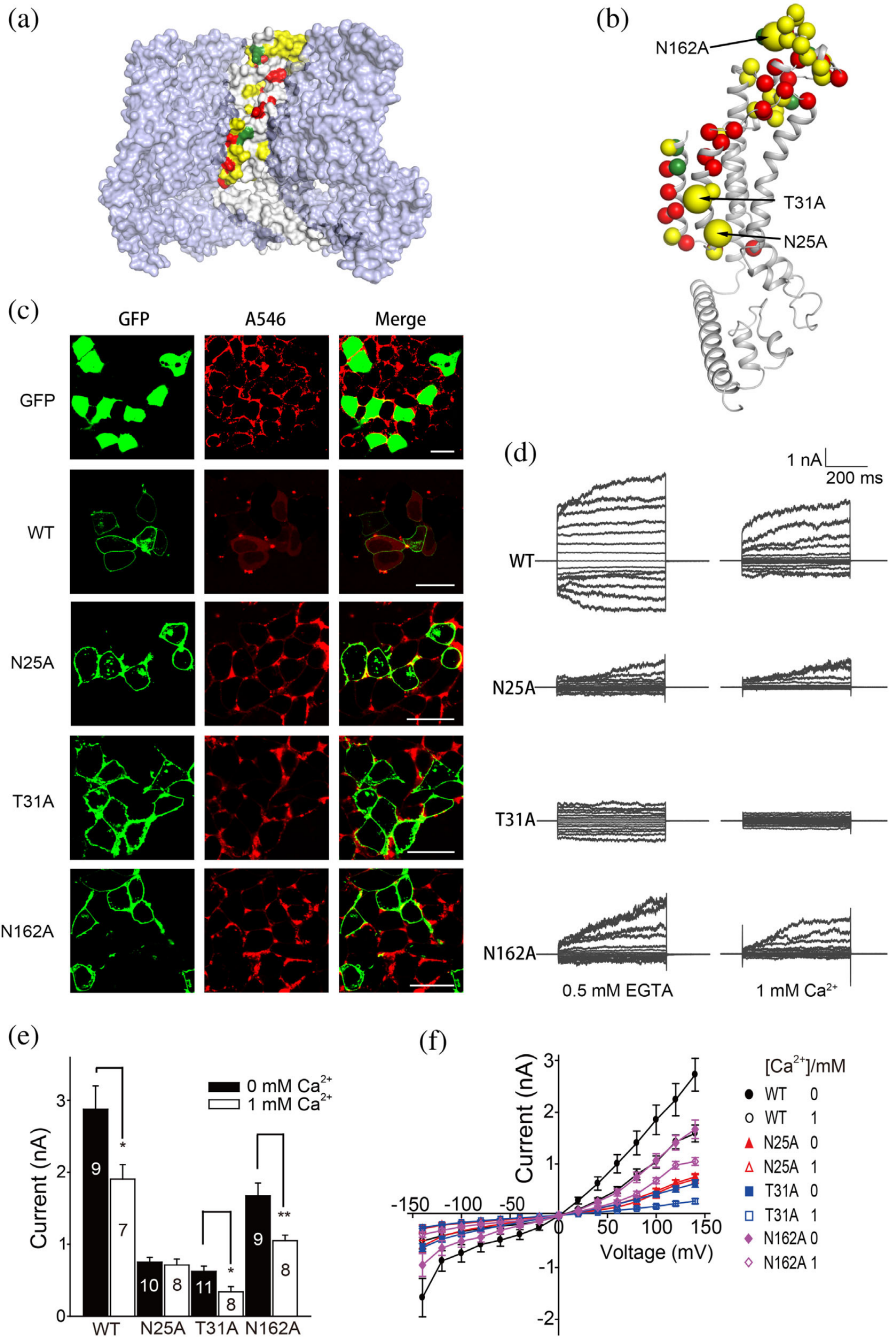
Mutation site distribution and characterization. (a) Distribution of all mutated sites in the interior of the hemichannel. Since the resolution in the S0 and S1 regions is low, assignment of the mutations in the model is tentative. (b) Position of the mutation sites in one protomer. The effects of the mutation are classified into three groups (see main text): the wild‐type (WT)‐like group is colored green, the “affected” group yellow, and the “disrupted” group red. (c) The Alexa 546 uptake assay. Fluorescence intensities of the *Ce*CLHM1‐green fluorescent protein (GFP) fusion protein (green) and of the Alexa 546 dye (red, A546) were recorded from cells expressing *Ce*CLHM1 variants in the presence or absence of extracellular Ca^2+^ to inhibit or activate the channel opening, respectively. Left: GFP‐CLHM1‐expressing cells were in the presence of extracellular Ca^2+^ (1 mM). Middle: Cells were incubated with ethylenediaminetetraacetic acid (EDTA) (50 mM) for 3 min to remove extracellular Ca^2+^. Right: Merged fluorescence images. (d) Representative current–voltage relationships obtained by applying 500 ms voltage pulses ranging from +140 to −140 mV from a holding potential of 0 mV (20 mV steps) to cells expressing WT *Ce*CLHM1, N25A, T31A, or N162A. Currents were recorded in the presence of 0.5 mM EGTA (0 mM Ca^2+^) and 1 mM Ca^2+^ introduced to the extracellular solution. (e) Statistical analysis of average current amplitude at +140 mV in 0.5 mM EGTA (i.e., 0 mM [Ca^2+^]_o_) and 1 mM [Ca^2+^]_o_ conditions for cells expressing WT CeCLHM1, N25A, T31A, or N162A. Two‐tailed paired *t* tests were applied to calculate *p* values for comparisons (*p < .05; **p < .01). Data are presented as mean ± *SEM*. The number of measurements (*n*) is indicated above the bar. (f) Current–voltage relationships of the data measured with cells expressing WT, N25A, T31A, or N162A *Ce*CLHM1 proteins, assayed either at 0 or 1 mM [Ca^2+^]_o_. The data are presented as mean ± *SEM*. Biologically independent experiments were performed for WT *C*eCLHM1, N25A, T31A, or N162A (*n* ≥ 7)

To identify critical amino acid residues involved in the gating mechanism, we introduced a total of 57 point mutations at a series of positions lining the pore lumen surface of the *Ce*CLHM1 channel (Figures 5a and S1), and analyzed their effects using dye‐uptake^21^ and electrophysiological assays. The mutation sites were concentrated in the S0 and S1 regions as well as the ECD where we expected the gating structural elements reside.^3^, ^22^ While most of the mutations were substituted into Ala, five acidic residues were mutated to Arg instead of Ala.

In order to analyze their cellular localization, we expressed the GFP‐fusion forms of *Ce*CLHM1 variants in HEK293T cells. First, the plasma‐membrane localization of wild‐type (WT) *Ce*CLHM1 was confirmed by confocal microscopy (green spots in Figure 5c), whereas the cells expressing GFP alone (negative control) lacked such specific localization. Interestingly, while the heterologously expressed *Ce*CLHM1 proteins were mainly located on the plasma membrane in most cells, a portion of the cells expressed the protein variants and WT proteins internally. In this small portion of cells, the intracellularly expressed *Ce*CLHM1 variants appeared to be colocalized with the marker of mitochondrial membrane (Mito‐tracker), not, however, with our lysosomal marker (Figure S8a). A transgenic line of *C. elegans* expressing *clhm‐1‐gfp* showed that *Ce*CLHM1‐GFP was predominantly distributed along the plasma membrane of neurons as well as muscle cells (Figure S8b), while no mitochondrial expression was detected. Thus, we cannot rule out the possibility that mitochondrial localization of *Ce*CLHM1 in HEK293T cell was an artifact caused by overexpression.

Next, we analyzed the channel activity of *Ce*CLHM1 using the dye‐uptake assay. As shown in Figures 5c and S9, in the HEK293T cells in which *Ce*CLHM1‐GFP was primarily expressed on the plasma membrane, the WT channel started to uptake the red fluorescent dye Alexa 546 from the environment upon removing extracellular Ca^2+^. In contrast, the negative control, where GFP was expressed by itself, failed to cause uptake of the dye upon reduction of [Ca^2+^]_o_. These observations are consistent with a previous report showing that the mouse CALHM1 channel facilitates the transport of a variety of dye molecules with molecular weights up to 760 Da.^21^

Importantly, this dye‐uptake assay allows for quick identification of residues essential for channel gating. Our 57 point mutations showed highly diverse effects in such a dye‐uptake assay, which we categorized into three major groups, according to their expression and dye absorption behaviors (Figure S9, Table S3). The first group, called WT‐like group, comprises five mutants, namely S4A, N6A, E136R, K163A, and R169A. All variants in this group showed normal expression levels as well as localization, and transported the dye in a manner similar to WT. None of these positions are conserved in human CALHMs, and all are (partially) solvent‐exposed in the current channel structure.

The second group, called “affected” (or loss‐of‐function) group, comprises 28 variants that were expressed well, but failed to transport the dye in response to the reduction of [Ca^2+^]_o_. In agreement with previous reports,^2^, ^14^ we found that the D125R mutant of *Ce*CLHM1 showed normal localization but was unable to absorb the red dye. In addition, all eight point mutants in the extracellular helix‐2 (Residues 150–160) belong to this loss‐of‐function group.

The third group, called “disruptive” group, comprises 24 variants that showed several types of defects in expression (such as K41A, C46A, and E106R) or localization (such as C48A, Y50A, and C131A). These defects presumably caused by disrupting the channel assembly and/or trafficking. In certain cases, even the cell morphology was affected, possibly because of improper opening of the channel (i.e., gain‐of‐function phenotype). For instance, all mutations around Loop‐2 and disulfide bond‐disrupting mutations in ECD belong to this group. Since all three categories contain charge‐varying mutations, it is unlikely that our screening results were the effects of changes in charge state of the channel on the diffusion of the negatively charged Alexa 546 dye.

### 2.4 *Electrophysiological analysis*

To investigate their channel activities, we performed whole‐cell electrophysiological analysis on selected *Ce*CLHM1 variants in CHO cells, a cell type characterized by low endogenous ionic currents.^23^ Native CHO cells and the GFP‐transfected cells serving as negative controls were first measured for background currents (Figure 2). Their amplitudes were 0.47 ± 0.04 nA (mean ± *SEM*) and 0.21 ± 0.04 nA per assay at +140 mV (Figure 2d), respectively, under conditions with ethylene glycol bis(2‐aminoethyl)tetraacetic acid (EGTA) to minimize [Ca^2+^]_o_; notably, the currents changed little in response to a change in [Ca^2+^]_o_. In contrast, the CHO cells expressing WT *Ce*CLHM1 protein prominently generated an outward current of 2.88 ± 0.33 nA at +140 mV under the condition with EGTA (Figure 2). This current was significantly higher than the background current of the negative controls. Consistent with previous studies,^14^ the channel exhibits clear voltage‐dependent activation and [Ca^2+^]_o_‐dependent inhibition (Figure 2). Thus, the recombinant WT *Ce*CLHM1 protein expressed on the plasma membrane of CHO cells formed active channels displaying behavior that is characteristic for members of the CALHM family.

Next, we applied whole‐cell electrophysiological analysis to selected variants from the “affected” group. For instance, cells expressing the N25A, T31A, or N162A variant exhibited loss‐of‐function phenotypes in the dye uptake assay (Figure 5). In agreement with this finding, the cells expressing these three mutant proteins exhibited currents of 0.75 ± 0.06 nA (N25A), 0.62 ± 0.07 nA (T31A), or 1.67 ± 0.18 nA (N162A) under the condition with EGTA, which was considerably lower than that of WT (2.88 ± 0.33 nA) (Figure 5d,e). In the presence of 1 mM Ca^2+^, whole‐cell currents were measured as 0.71 ± 0.08 nA (N25A), 0.28 ± 0.05 nA (T31A), and 1.05 ± 0.08 nA (N162A), respectively, which were also significantly lower than the current measured for cells expressing the WT channel (1.91 ± 0.20 nA) (Figure 5d). Furthermore, the currents of T31A and N162A channels were both significantly inhibited by 1 mM [Ca^2+^]_o_ as the WT channel did (Figure 5f,g). In contrast, the current of N25A appeared to be similar under conditions with or without extracellular Ca^2+^, suggesting that this mutant becomes insensitive to the regulation by [Ca^2+^]_o_.

## 3 DISCUSSION

Here, we solved the three‐dimensional structures of the recombinant *Ce*CLHM1 channel in its open form and analyzed the functional roles of a series of pore‐lining residues through a dye‐uptake assay and an electrophysiological experiment. Whereas the *Ce*CLHM1 channel appears to show an overall architecture similar to CALHM (hemi‐)channels recently reported,^10^, ^11^, ^12^, ^13^ we observed a few major differences between these channels (Figure S10). First, *Ce*CLHM1 exhibits a double‐layered symmetrical dimer of decameric hemichannels in a tail‐to‐tail packing fashion, in contrast to the more popular head‐to‐head packing. Nevertheless, a recently reported hCALHM4 structure also showed such a tail‐to‐tail packing (Figure S11), suggesting that it may not be an isolated phenomenon. The interface in our double‐layered *Ce*CLHM1 channel buries 3,800 Å^2^ of SAS area. Although this value appears to be rather small considering that 20 subunits participate in this docking, it is significantly larger than the SAS area calculated for the hCALHM2 gap junction structure (6UIX; 1,800 Å^2^). The head‐to‐head type of docking is likely to result in an intercellular gap junction between two adjacent cells, unless glycosylation prevents such docking process. However, such a putative gap junction seems difficult to consolidate with the known functions of the CALHM channels in delivering the neurotransmitter ATP in atypical synapses.^9^, ^24^ In this case, the paracrine ATP should be exported into the synapse gap to activate specific receptors (e.g., P2Xs) on the surface of the postsynaptic cell to evoke a cellular response,^9^, ^16^ instead of being directly injected into the postsynaptic cell. However, the tail‐to‐tail type of docking observed in the current study may indicate that a different type of gap junction exists between the plasma membrane and certain organelles. In agreement with this hypothesis, we found that *Ce*CLHM1 colocalizes with mitochondrial markers in HEK293 cells (Figure S8a). The expression of *Ce*CLHM1‐GFP fusion in *C. elegans* reveals that the *Ce*CLHM1 protein is widely distributed in the sensory neurons and muscle cells. We noted that the fusion of GFP at the C‐terminal end of the *Ce*CLHM1 protein is unfavorable for formation of the double‐layered channel (Figure S3). Thus, further in situ studies on the subcellular localization of GFP‐free *Ce*CLHM1 protein, for example through immunochemical assays using specific antibodies, are necessary to confirm the precise mitochondrial colocalization of *Ce*CLHM1. If *Ce*CLHM1 indeed implements certain types of intracellular gap junction, it remains to be investigated how the hemichannels on the organelle membrane are regulated in cooperation with the hemichannels localized on the plasma membrane to ensure synergetic opening. Although we cannot rule out that the observed double‐layered docking simply is an artifact of in vitro studies, the rotational symmetry of the hemichannel may also facilitates in vivo dimerization by reducing the entropy penalty during the docking of one hemichannel onto another, provided that the channels are also expressed on the organelle membrane. Since the regions involved in neither head‐to‐head nor tail‐to‐tail type of channel packing are conserved among the CALHM family members (Figure S1), it is likely that large number of protomers in each hemichannel promotes the frequently observed dimerization.

The second feature of *Ce*CLHM1 is its ability to establish multiple forms of hemichannels. We observed a mixture of nonamers, decamers, and undecamers, with decamers being the dominant species. In comparison, the reported CALHM channels also take forms ranging from octamer to dodecamer, but are mostly present in one or two forms for each given CALHM/CLHM species (Figure S10, Table S1). In fact, in a recent report on *Ce*CLHM1 structure,^11^ only the nonameric form was observed, without dimerization of the hemichannel. In this previous study, the protein was purified using amphipol PMAL‐C8, rather than detergent (e.g., LMNG in our case). Since different CALHM proteins appear to show preference for different oligomeric forms, it is debated whether the TM domain or the cytoplasmic domain of the protomers determines the final oligomerization state.^11^, ^12^ The different forms of *Ce*CLHM1 further suggest that the solubilization technique is one more factor that is likely to perturb the oligomeric states of the channel. Moreover, the coexistence of multiple forms indicates that the protomers are of intrinsic structural flexibility to accommodate slightly different intersubunit packing. It is likely that a given CALHM/CLHM channel maintains a certain distribution of multiple forms of oligomeric states and even heteromeric states^6^ under in vivo conditions. Furthermore, unlike in canonical channels that are composed of small number of subunits, opening of a CALHM/CLHM channel may result from collective (though not necessarily synchronized) structural adjustment of its protomers. Therefore, the activity of a CALHM/CLHM channel is unlikely strictly depend on the number of its protomers.

The third feature of *Ce*CLHM1 was identified at its N‐terminal region. Consistent with the secondary structure prediction result,^25^ the N‐terminal region of each *Ce*CLHM1 protomer forms an α‐helix called S0. In our *Ce*CLHM1 structure, both S0 and S1 maintain a vertical position, lining the inner surface of the pore. A similar S0 helix has been observed in hCALHM4 (Figure S11; Reference 10) and killifish CALHM1 (Reference 11) channels, although in both cases the N‐terminal end projects toward the pore central axis. The central pore of the *Ce*CLHM1 channel appears to be open at a diameter of ~40 Å at the narrowest site, large enough for transporting ATP as well as ions. On the basis of these varied positions of the N‐terminal helices in the recently reported CALHM/CLHM structures, a consensus is emerging as that the channel may change its conductance by adjusting the relative position of S0 as well as S1 to the central axis of the pore. For instance, the S1 helix in each subunit of the hCALHM2 channel is lifted up due to conformational change induced by a bound RuR molecule, and thus the inner diameter of the channel is reduced from 40 to 23 Å (Reference 13). However, the RuR‐binding site observed in the hCALHM2 structure is not conserved in *Ce*CLHM1 (Figure S1), suggesting that this inhibitor may interact with a different binding site in *Ce*CLHM1. Interestingly, complete truncation of the S0 helix in the octameric channel of killifish CALHM1, which shows the narrowest pore size (16 Å in diameter) among reported CALHM/CLHM structures, failed to abolish the regulation of the channel by [Ca^2+^]_o_.^11^ This observation is in sharp contrast to the effects of our point mutation within the S0 region. One way to consolidate the discrepancy is to hypothesize that, upon truncation of S0, the enlarged central pore is compensated by more lipid molecules enclosed within the channel pore. In support of this idea, lipid bilayer‐like densities were observed inside the hydrophobic channel pores of a number of recently reported CALHM structures.^10^, ^12^ The two layers of positive charges in the interior of the pore observed in our *Ce*CLHM1 structure (Figure S7) is consistent with this notion. As different pores in their resting state may accommodate varying amounts of lipid molecules, the relative change in pore size during the activation process (rather than its absolute diameter) may not only affect the conductance but also influence the open‐state probability of a given channel. Such increase in the pore size should be sufficiently large to permit permeation of ATP as a major in vivo substrate.

In conclusion, our structure of *Ce*CLHM1 confirmed that multiple subunit assembly is a common structural feature of channels of the CALHM family and that the N‐terminal two helices are most likely to function as a shutter to control the opening of the channel. The tail‐to‐tail stacking of two hemichannels suggests that the CALHM/CLHM family represents a new type of gap junctions. Our structural and functional analyses provide new insights critical for a better understanding of the roles of crucial amino acid residues on the pore lumen surface.

## 4 MATERIALS AND METHODS

### 4.1 *Construct design, protein expression, and purification*

The gene encoding full‐length CLHM1 of *C. elegans* (UniProtKB number: Q18593) was cloned into a modified pEG BacMam vector followed by GFP and a C‐terminal His_10_‐tag as well as a preceding PreScission protease cleavage site. This construct was used to express *Ce*CLHM1 protein in HEK293F cells using the BacMam method.^26^ Briefly, a bacmid plasmid containing the *clhm‐1* gene was acquired from transforming *Escherichia coli* DH10bac cells, in accordance with the manufacturer's instructions (Bac‐to‐Bac; Invitrogen, Carlsbad, CA). Next, we obtained the baculoviruses through transfecting *Spodoptera frugiperda*‐9 cells with the bacmid plasmid. After 72 hr of amplification, the baculoviruses were used to transfect HEK293F cells. When the cells grew to a density of 2.5 × 10^6^ cells/ml, the baculoviruses were added (5% vol/vol) to initiate transfection. After 8 hr, 5 mM sodium butyrate was added and growth was continued at 37°C until 48 hr posttransfection.

For purification, the pellet from 800 ml of HEK293F cell culture was lysed with a Dounce homogenizer in Buffer A (50 mM HEPES [pH 7.5], 150 mM NaCl, 8 μM BPY [Macklin, Shanghai, China], and protease inhibitor cocktail) at 4°C. The cell lysate was supplemented with 2 μg/ml (final concentration) iodoacetamide (Sigma, St. Louis, MO) and incubated for 30 min to block cysteine residues. Next, 1% (wt/vol) LMNG (Anatrace, Santa Clara, CA) was added to solubilize the membrane and incubated at 4°C for 2 hr. The insoluble debris was removed by ultracentrifugation at 10,000*g* for 30 min. To purify the protein, the supernatant was incubated with cobalt resin (Thermo Fisher Scientific, Waltham, MA) and 20 mM imidazole at 4°C for 2 hr. The resin was then washed with 20 column volumes of Buffer B (Buffer A plus 25 mM imidazole and 0.05% LMNG). To cleave off the GFP and His_10−_tag, the washed resin was incubated overnight with PreScission protease. The protein was then eluted with Buffer B. After concentration using 100‐kDa cut‐off concentrator (Millipore, Bedford, MA), the purified protein was injected into a Superose‐6 column (GE Healthcare, Uppsala, Sweden) equilibrated with buffer C (50 mM HEPES [pH 7.5], 150 mM NaCl, 0.02% LMNG, and 8 μM BPY) for SEC purification. Finally, the peak fraction was collected and concentrated to 1.5–2.0 mg/ml for EM grid preparation.

### 4.2 *Cryo‐EM sample preparation and data acquisition*

Samples of *Ce*CLHM1 (4 μl) with or without BPY (8 μM) were applied to a glow‐discharged Quantifoil R2/1 copper grid (Quantifoil Micro Tools GmbH) and vitrified by plunge‐freezing in liquid ethane using a Vitrobot Mark IV (Thermo Fisher Scientific) with a blotting time of 3 s and blotting force of 2 N. The *Ce*CLHM1 data were collected on a Titan Krios microscope operated at 300 kV and the *Ce*CLHM1‐BPY data were collected on a Talos Arctica microscope operated at 200 kV, respectively, at the Center of Biological Imaging at the Institute of Biophysics (CBI‐IBP), CAS. Both microscopes were equipped with a field emission gun and a Gatan K2 Summit direct electron camera in a superresolution mode. The calibrated magnification was ×22,500, corresponding to a pixel size of 1.04 Å for the Titan Krios and 1.00 Å for the Talos Arctica detector, respectively. The automated software SerialEM was used to collect 7,972 movies for *Ce*CLHM1 and 3,930 movies for the *Ce*CLHM1‐BPY sample with a defocus range of 1.8–2.3 μm. Each movie with 10 s exposure comprised 32 subframes, amounting to a total dose of 60 electrons Å^−2^ s^−1^.

### 4.3 *Image processing*

Data processing flowcharts of the two *Ce*CLHM1 and *Ce*CLHM1‐BPY datasets were nearly identical. Micrograph movie stacks were corrected for beam‐induced motion using MotionCor2.^27^ The contrast transfer function parameters for each dose‐weighting image were determined using Gctf.^28^ Particles were initially autopicked using Gautomatch with templates from negative staining results and extracted by placing a shrunk 150‐pixel by 150‐pixel box around each potential target. After iterative 2D class averaging in cryoSPARC^29^ and RELION3,^30^, ^31^ the classification results indicated both *Ce*CLHM1 and *Ce*CLHM1‐BPY samples present a gap‐junction‐like channel shape with various polymerization and stacking patterns, including 9‐mer, 10‐mer, and 11‐mer polymerization from the top‐view, and a double‐stack of hemichannels from the side view (Figures S4c and S5c). To determine the overall structure of the gap‐junction‐like channel, particles were manually selected from 2D class average results and subjected to 3D classification in Relion with a D10 symmetry superimposed. The particles showing good 3D class averages were exported into cryoSPARC and subjected to a nonuniform refinement, which produced the final density maps with an overall resolution of 4.18 Å for *Ce*CLHM1 and 3.73 Å for *Ce*CLHM1‐BPY, as given by the *Fourier* shell correlation 0.143 criterion (Figures S4c and S5c). To reveal the polymerization of the hemichannels, two hemichannel particles were recentered and extracted from each of the gap‐junction‐like channel particle^32^ by calculating a vector from the center of gap‐junction‐like channel to centers of the two hemichannels. The reextracted hemichannels were then subjected to another 3D classification without symmetry constraint. Particles from the classes with similar features were merged for further 3D refinement, which yielded different polymerized maps for both *Ce*CLHM1 and *Ce*CLHM1‐BPY, that is, nonamer, decamer, and undecamer (Figures S4c and S5c). Unexpectedly, nonamer and undecamer maps of *Ce*CLHM1‐BPY were blurred, implying that the decamer was the predominant form upon addition of BPY. To further solve the N‐terminal structure of *Ce*CLHM1, we applied local resolution estimation and local filtering in cryoSPARC^33^ to both *Ce*CLHM1 and *Ce*CLHM1‐BPY datasets, which produced the reasonable N‐terminal densities.

### 4.4 *Model building and refinement*

Before de novo modeling of *Ce*CLHM1, secondary and tertiary structure prediction were performed using PSIPRED^34^ and I‐TASSAR.^25^ Using the prediction as a reference, the structure was manually built using the COOT^35^ program according to the density map. Real space refinement was carried out using the Phenix.real_space_refine software utility,^36^ with restraints on secondary structures as well as Ramachandran distribution. Chimera^37^ and PyMOL^38^ were used for graphical visualization.

### 4.5 *Cell culture*

HEK293T cells were grown in Dulbecco's Modified Eagle's Medium (DMEM) (Invitrogen), supplemented with 10% (vol/vol) fetal bovine serum (Gibco/Invitrogen). Cells were grown in a 37°C incubator with a 5% CO_2_ humidified environment and treated twice a week with 0.05% (wt/vol) trypsin and 0.5 mM ethylenediaminetetraacetic acid (EDTA) in phosphate‐buffered saline (PBS) solution. For gene transfection, CHO cells were transferred to glass coverslips coated with poly‐L‐lysine. After the cells reached 50–70% confluence, the *Ce*CLHM1‐GFP protein was transiently expressed with polyethyleneimine (PEI) at a plasmid:PEI ratio of 1:3 (wt/wt). The cells were used for electrophysiological studies 16–36 hr after the transfection.

### 4.6 *Alexa 546 dye uptake experiment*

For all imaging experiments, HEK293T cells were plated on glass coverslips 1 day prior to transfection. *Ce*CLHM1‐GFP‐expressing cells were washed twice with PBS solution and used for imaging. Next, the extracellular Ca^2+^ was quickly removed by incubating the cells in 50 mM EDTA for 3 min to activate the channel. Subsequently, the solution was replaced by fresh PBS with 10 μM Alexa 546 (Life Technology, Waltham, MA) and 50 mM EDTA. The dye uptake process was recorded using a video camera.

### 4.7 *CHO cell electrophysiology*

CHO cells were grown in DMEM and F12 (Invitrogen) (1:1) supplemented with 10% (vol/vol) fetal bovine serum. Cells were grown in a 37°C incubator with a 5% CO_2_ humidified environment and treated twice a week with exposure to 0.05% (wt/vol) trypsin and 0.5 mM EDTA in PBS solution. For gene transfection, CHO cells were transferred to glass coverslips coated with poly‐l‐lysine. After the cells reached 50–70% confluence, the *Ce*CLHM1‐GFP was transiently expressed with PEI at a ratio of 1:3 (wt/wt).

Whole‐cell currents were recorded 16–36 hr after transfection. GFP‐positive cells were selected under a fluorescence microscope and used for subsequent patch‐clamp experiments. The pipette solution contained 130 mM KCl, 10 mM NaCl, 1 mM CaCl_2_, 11 mM EGTA, and 10 mM HEPES (pH 7.3, adjusted with KOH). Bath solutions contained 140 mM NaCl, 5.4 mM KCl, 1 mM MgCl_2_,10 mM glucose, 10 mM tetraethylammonium, and 10 mM HEPES (pH 7.4), and varying concentrations of Ca^2+^ as indicated in the results section. To deplete Ca^2+^ in the extracellular environment, EGTA (0.5 mM) was added to the bath solution to create a Ca^2+^‐free condition. Data were acquired using an EPC‐10 amplifier (HEKA, Germany) in whole‐cell mode at 5 kHz. Currents were filtered by an eight‐pole Bessel filter at 1 kHz and sampled at 5 kHz. Electrodes were made from thick‐walled B15023F glass (Wuhan Vitalsense Scientific Instruments, Wuhan, China). All experiments were performed at room temperature (21–24°C).

### 4.8 *Expression analysis of clhm‐1 in *C. elegans**

To generate P*clhm1*::*clhm1*::*gfp*, 2.2‐kb promoter and the entire open reading frame of *clhm‐1* were amplified from fosmid WRM0610dF07. The promotor sequence was inserted into the pPD49.26 backbone to generate P*clhm1*::*gfp*. The entire open reading frame of *clhm1* was then inserted into P*clhm1*::*gfp* by *Nhe*I and *Kpn*I. The correctness of all plasmids was confirmed by sequencing.

The construct P*clhm1*::*clhm1*::*gfp* (5 ng/μl) was coinjected with an *unc‐76* expression vector (p76‐16B) (50 ng/μl) and *Pnfya‐1::*(*mito*)*mAKAP1::mRFP* (5 ng/μl) into *unc‐76*(*e911*) animals. Both transgene lines were used for confocal microscopy analysis. *C. elegans* were cultured at 20°C on King Agar plates covered with *E. coli* OP50. CLHM1::GFP was mainly expressed at or near the plasma membrane of body wall muscle and sensory neurons, and GFP signals were not detectable in hypodermis cells and intestinal cells in larval and adult stages. These observations were in agreement with findings reported in a previous study.^14^

## CONFLICT OF INTEREST

The authors declare that there is no conflict of interest that could be perceived as prejudicing the impartiality of the research reported.

## AUTHOR CONTRIBUTIONS

Xuejun C. Zhang supervised the project. Ye Zhou and Weixin Yang initiated the project and purified the proteins. Youwang Wang, Weixin Yang, and Ping Zhu performed the cryo‐EM experiments and structure determination. Weixin Yang constructed the mutations. Jianli Guo and Zhenfeng Liu performed the electrophysiology studies and data analysis. Weixin Yang, Lingli He, and Hui Zheng performed cell biology experiments. Weixin Yang, Youwang Wang, Zhenfeng Liu, Ping Zhu, and Xuejun C. Zhang analyzed the structure and wrote the manuscript. All of the authors contributed to manuscript preparation.

## Supporting information


**Appendix** **S1**: Supporting Information

## Data Availability

The coordinates of CeCLM1 have been deposited in the PDB database with accession code 6LOM. The cryo‐EM map is accessible with EMD number EMD‐0938.
