# Supplementary material for: Cryo‐electron microscopy structure of CLHM1 ion channel from Caenorhabditis elegans
Source: Protein Sci. 2020 Jun 30;29(8):1803–15. doi: 10.1002/pro.3904 (PMC7380676; doi:10.1002/pro.3904)
Supplement: Supplementary file 1 — Appendix S1: Supporting Information [file PRO-29-1803-s001.pdf]

Cryo-EM structure of CLHM1 from *Caenorhabditis elegans* (Supplemental Material)

Supplemental figures

Figure S1. Sequence homology of CeCLHM1 with homologs

a | Sequence alignment of *C. elegans* CLHM1 (NP_495403.2) with human CALHM1 (NP_001001412.3), CALHM2 (pdb_6UIV), CALHM3 (NP_001123214.1), and mouse CALHM1 (NP_001074740.1). Conserved residues are highlighted in colors, blue for absolute identity and green for those of conserved properties. Secondary structures in *Ce*CLHM1 are marked on top, and omitted parts are marked on dash lines. Residues involved in RUR binding in hCALHM2 are marked with hollow diamonds. Solid circles denote mutation sites in *Ce*CLHM1. In particular, green is for the WT-like group; yellow for the ‘affected’ group; and red for the ‘disrupted’ group. The sequences were alignment with the Clustal Omega program (*1*).

b | More sequence alignment of the CALHM family members from different species.

c | Phylogenetic tree of the CALHM family. Related sequence IDs Include human CALHM4 (NP_001353007.1); human CALHM5 (NP_714922.1); human CALHM6 (NP_001010919.1); Rat CALHM1 (NP_001102638.1); Chicken CALHM1 (XP_015144219.1); *C. remanel* (worm) CLHM1 (XP_003113485.1); *D. pachys* *（worm）* WR25_05457 (PAV75100.1); *S. carpocapsae* (worm) H_L596_002764 (TMS35340.1); *P. pacificus* (worm) CLHM1 (PDM83315.1); *S. mimosarum* (spider) FAM26F (KFM62005.1); *X. laevis* (frog) FAM26E (XP_018120870.1); *H. armiger* (bat) FAM26F (XP_019523821.1).

(Fig. S1a)

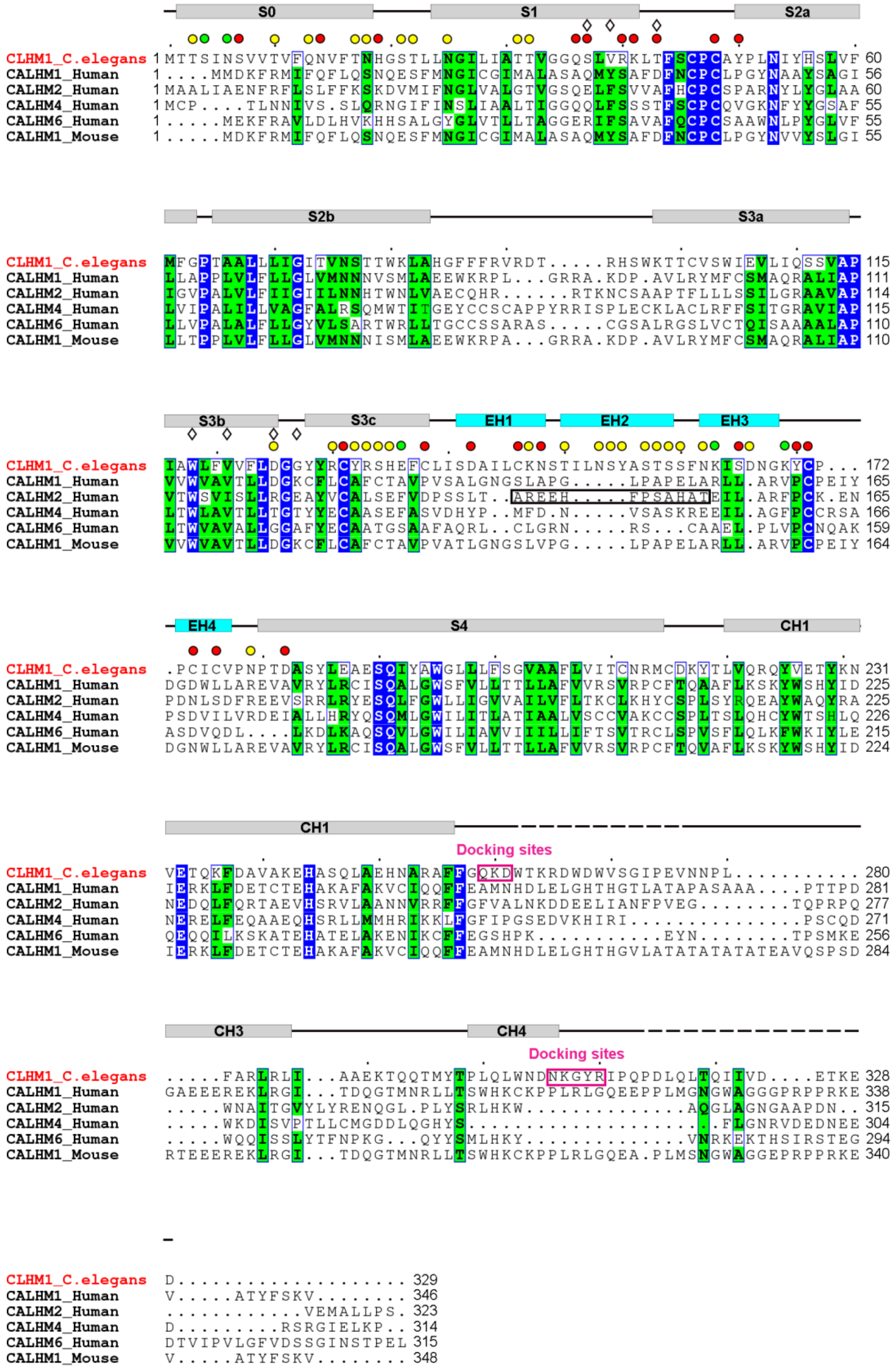
S0
S1
S2a
CLHM1_C.elegans 1 MTTSINSVVTVFQNVFTNHGSTLLNGILIATTVGGQSLVRKLTFSCPCAYPLNIYHSLVF 60
CALHM1_Human 1 ....MMDKFRMIFQFLQSNQESFMNGICGIMALASAQMYSAFDFNCPCLPGYNAAYSAGI 56
CALHM2_Human 1 MAALIAENFRFLSLFFKSKDVMIFNGLVALGTVGSQELFSVVAFHCPCSPARNYLYGLAA 60
CALHM4_Human 1 MCP....TLNNIVS.SLQRNGIFINSLIAALTIGGQQLFSSSTFSCPCQVGKNFYYGSAF 55
CALHM6_Human 1 .....MEKFRAVLDLHVKHHSALGYGLVTLLTAGGERIFSAVAFQCPCSAAWNLPYGLVF 55
CALHM1_Mouse 1 .....MDKFRMIFQFLQSNQESFMNGICGIMALASAQMYSAFDFNCPCLPGYNVVYSLGI 55
S2b
S3a
CLHM1_C.elegans MFGPTAALLLIGITVNSTTWKLAHGFFFRVRDT.....RHSWKTTCVSWIEVLIQSSVAP 115
CALHM1_Human LLAPPLVLFLLGLVMNNNVSMLAEEWKRPL...GRRA.KDP.AVLRYMFCSMAQRALIAP 111
CALHM2_Human IGVPALVLFIIGIILNNHTWNLVAECQHR......RTKNCSAAPTFLLLSSILGRAAVAP 114
CALHM4_Human LVIPALILVAGFALRSQMWTITGEYCCSCAPPYRRISPLECKLACLRFFSITGRAVIAP 115
CALHM6_Human LLVPALALFLLGYVLSARTWRLLTGCCSSARAS.....CGSALRGSLVCTQISAAAALAP 110
CALHM1_Mouse LLTPPLVLFLLGLVMNNNISMLAEEWKRPA...GRRA.KDP.AVLRYMFCSMAQRALIAP 110
S3b
S3c
EH1
EH2
EH3
CLHM1_C.elegans IAWLFVVFLDGGYYRCYRSHEFCLISDAILCKNSTILNSYASTSSFNKISDNGKYCP... 172
CALHM1_Human VVWVAVTLLDGKCFLCAFCTAVPVSALGNGSLAPG.....LPAPELARLL.ARVPCPEIY 165
CALHM2_Human VTWSVISLLRGEAYVCALSEFVDPSSLT..AREEH.....FPSAHATEIL.ARFPCK.EN 165
CALHM4_Human LTWLAVTLLTGTYYECAASEFASVDHYP..MFD.N.....VSASKREEIL.AGFPCCRSA 166
CALHM6_Human LTWVAVALLGGAFYECAATGSAAFAQRL..CLGRN.....RS...CAAEL.PLVPCNQAK 159
CALHM1_Mouse VVWVAVTLLDGKCFLCAFCTAVPVATLGNGSLVPG.....LPAPELARLL.ARVPCPEIY 164
EH4
S4
CH1
CLHM1_C.elegans .PCICVPNPTDASYLEAESQIYAWGLLLFSGVAAFLVITCNRMCDKYTLVQRQYVETYKN 231
CALHM1_Human DGDWLLAREVAVRYLRCISQALGWSFVLLTTLLAFVVRSVRPCFTQAAFLKSKYWSHYID 225
CALHM2_Human PDNLSDFREEVSRRLRYESQLFGWLLIGVVAILVFLTKCLKHYCSPLSYRQEAYWAQYRA 225
CALHM4_Human PSDVILVRDEIALLHRYQSQMLGWILITLATIAALVSCCVAKCCSPLTSLQHCYWTSHLQ 226
CALHM6_Human ASDVQDL....LKDLKAQSQVLGWILIAVVIIILLIFTSVTRCLSPVSFLQLKFWKIYLE 215
CALHM1_Mouse DGNWLLAREVAVRYLRCISQALGWSFVLLTTLLAFVVRSVRPCFTQVAFLKSKYWSHYID 224
CH1
Docking sites
CLHM1_C.elegans VETQKFDAVAKEHASQLAEHNARAFFGQKDWTKRDWDWVSGIPEVNNPL........... 280
CALHM1_Human IERKLFDETCTEHAKAFAKVCIQQFFEAMNHDLELGHTHGTLATAPASAAA....PTTPD 281
CALHM2_Human NEDQLFQRTAEVHSRVLAANNVRRFFGFVALNKDDEELIANFPVEG........TQPRPQ 277
CALHM4_Human NERELFEQAAEQHSRLLMMHRIKKLFGFIPGSEDVKHIRI..............PSCQD 271
CALHM6_Human QEQQILKSKATEHATELAKENIKCFFEGSHPK...........EYN........TPSMKE 256
CALHM1_Mouse IERKLFDETCTEHAKAFAKVCIQQFFEAMNHDLELGHTHGVLATATATATATEAVQSPSD 284
CH3
CH4
Docking sites
CLHM1_C.elegans .....FARLRLI...AAEKTQQTMYTPLQLWNDNKGYRIPQPDLQLTQIIVD....ETKE 328
CALHM1_Human GAEEEREKLRGI...TDQGTMNRLLTSWHKCKPPLRLGQEEPPLMGNGWAGGGPRPPRKE 338
CALHM2_Human .....WNAITGVYLYRENQGL.PLYSRLHKW..............AQGLAGNGAAPDN.. 315
CALHM4_Human .....WKDISVPTLLCMGDDLQGHYS...................FLGNRVDEDNEE 304
CALHM6_Human .....WQQISSLYTFNPKG...QYYSMLHKY..............VNRKEKTHSIRSTEG 294
CALHM1_Mouse RTEEEREKLRGI...TDQGTMNRLLTSWHKCKPPLRLGQEA.PLMSNGWAGGEPRPPRKE 340
CLHM1_C.elegans D................... 329
CALHM1_Human V.....ATYFSKV........ 346
CALHM2_Human ....VEMALLPS. 323
CALHM4_Human D.........RSRGIELKP.. 314
CALHM6_Human DTVIPVLGFVDSSGINSTPEL 315
CALHM1_Mouse V.....ATYFSKV........ 348

(Fig. S1b, 1/2)

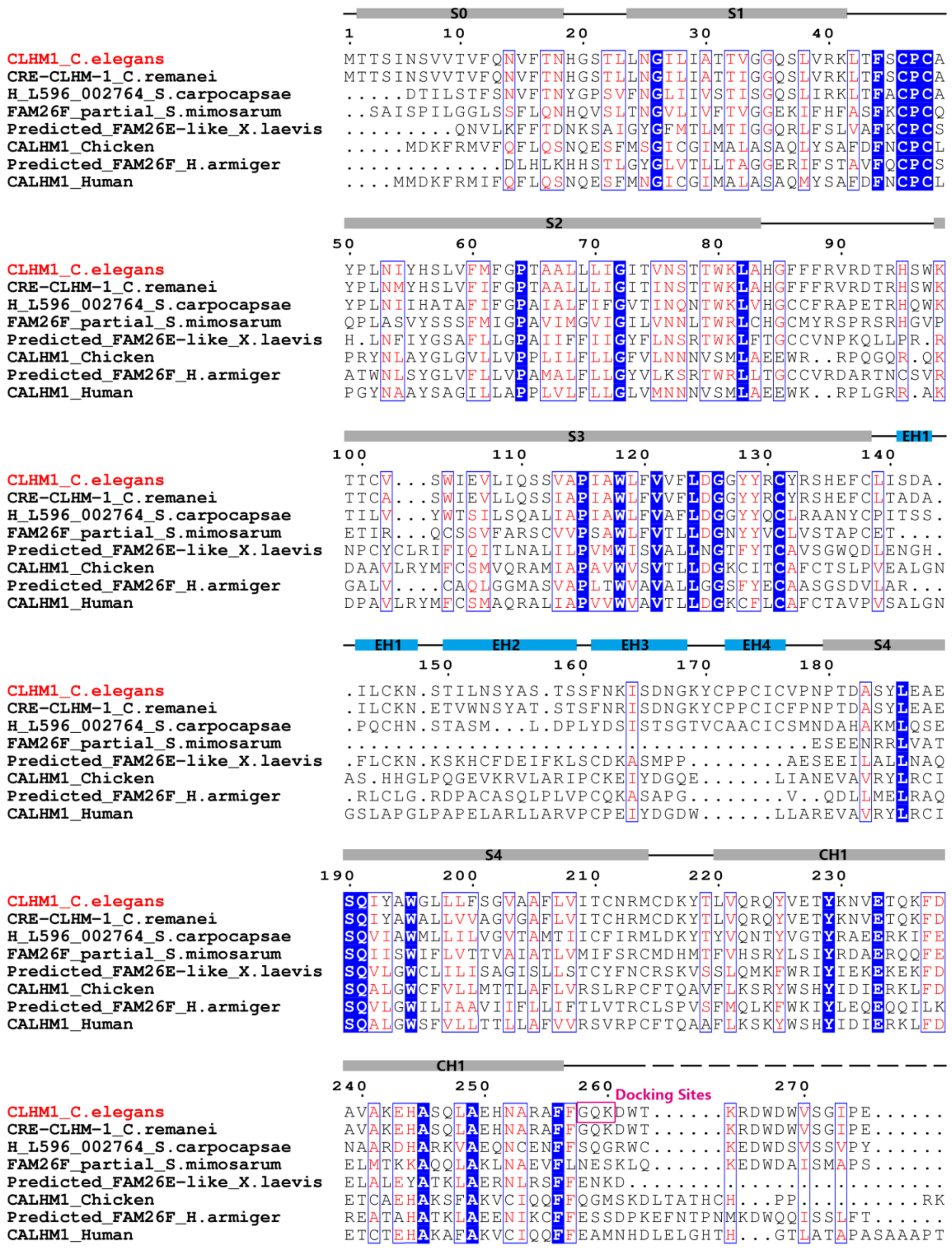
S0
S1
S2
S3
EH1
EH2
EH3
EH4
S4
CH1
Docking Sites
CLHM1_C.elegans
CRE-CLHM-1_C.remanei
H_L596_002764_S.carpocapsae
FAM26F_partial_S.mimosarum
Predicted_FAM26E-like_X.laevis
CALHM1_Chicken
Predicted_FAM26F_H.armiger
CALHM1_Human

(Fig. S1b, 2/2)

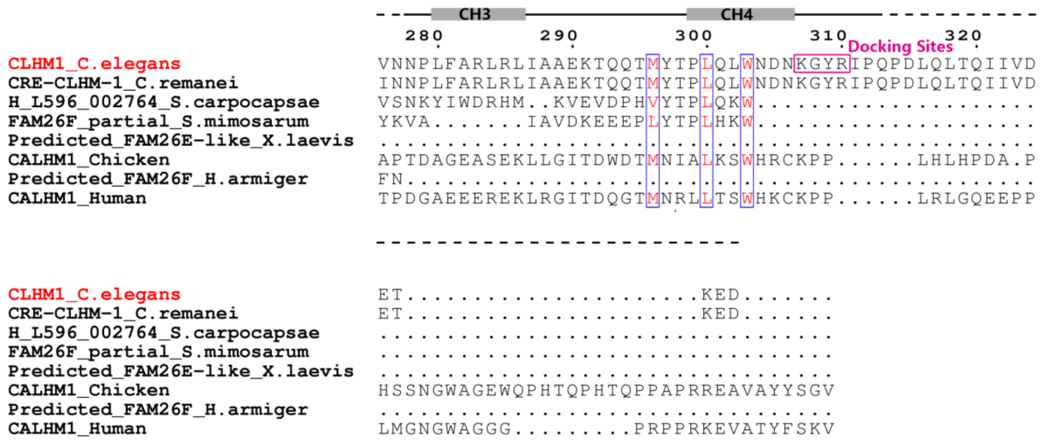

(Fig. S1c)

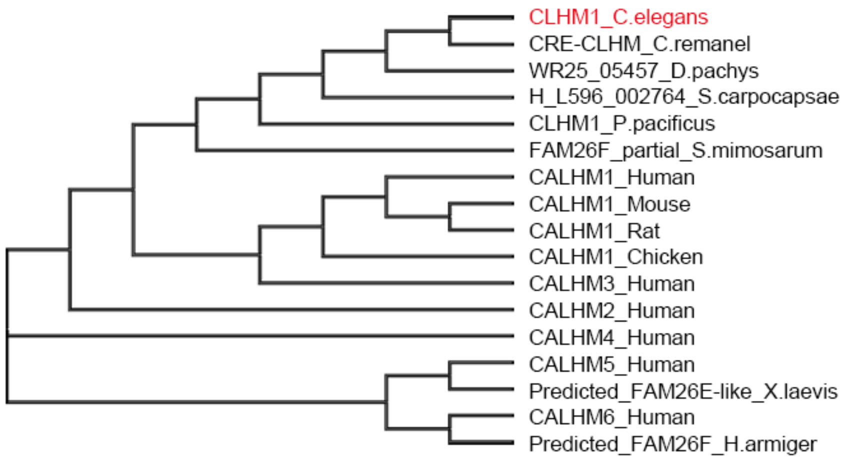

Figure S2. Sample preparation of recombinant CLHM1 from C. elegans

a | The CLHM1-GFP complex peak in the SEC elution profile is pointed with the red arrow.

b | The CLHM1 clipped GFP complex peak in the SEC elution profile is pointed with the red arrow.

c | The CLHM1-GFP and CLHM1 clipped GFP bands purified from HEK293T are shown in red boxes in a denaturing SDS gel.

d | Chemical structure of BPY.

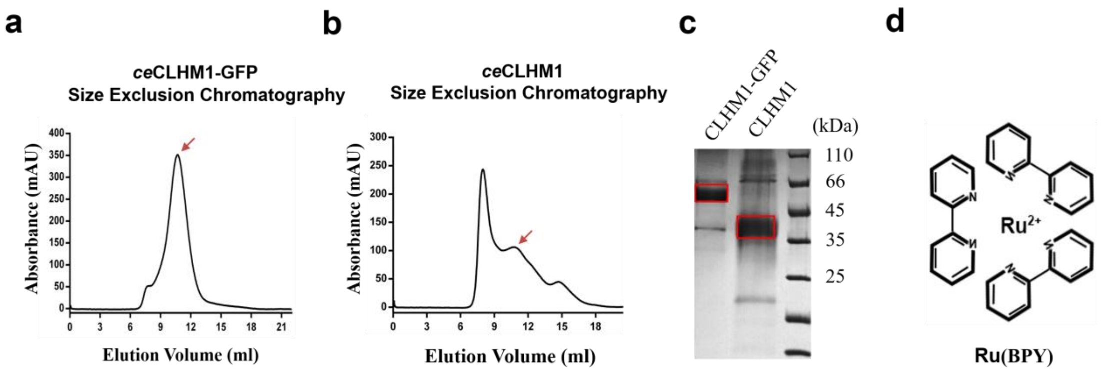

Figure S3. Initial data of CLHM1-GFP fusion complex

a | Representative negative stain micrograph of *Ce*CLHM1-GFP in LNMG. Selected particles are labelled with yellow arrows.

b | Representative cryo-EM micrograph of CeCLHM1-GFP in LNMG. Representative particles are circled.

c | 2D and 3D model with fuzzy density (GFP) of *Ce*CLHM1. Density of GFP is pointed with red arrows.

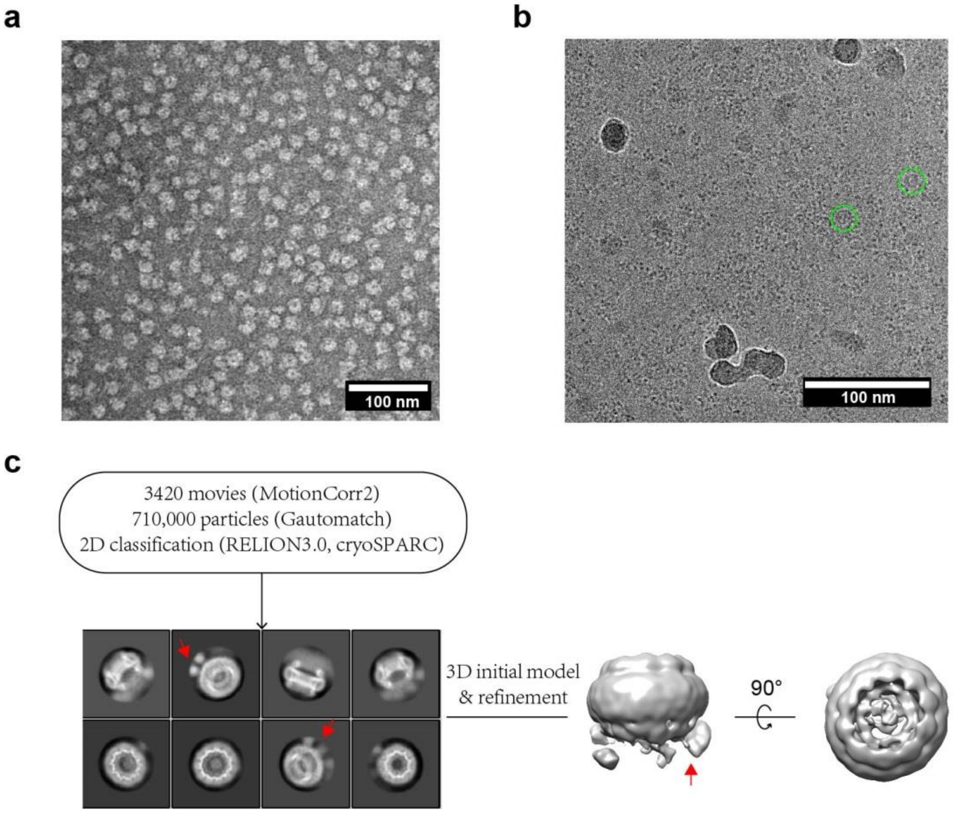

Figure S4. Structure determination of CeCLHM1 reconstituted in LMNG (native state).

a and b | Representative negative stain and cryo-EM micrograph of *Ce*CLHM1 in LNMG. Representative particles in b are circled.

c | Flowchart of cryo-EM micrographs processing for *Ce*CLHM1 and the FSC curves. The symmetries imposed during refinement were C9, C10, C11, and D10, individually. Due to severe orientation preference, the particle datasets shrank during classification process.

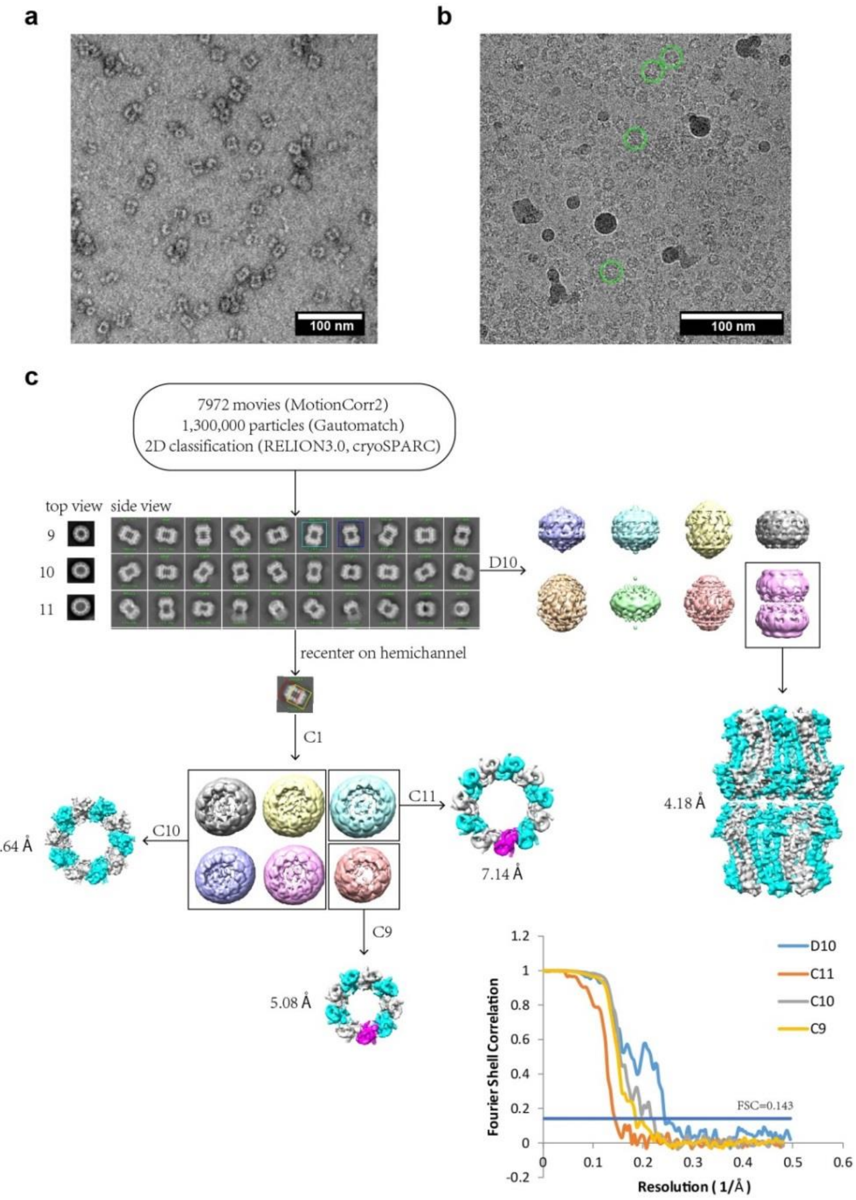

Figure S5. Structure determination of CeCLHM1 reconstituted in LMNG (with BPY).

a and b | Representative negative stain and Cryo-EM micrograph of *Ce*CLHM1 in LNMG. Representative particles in b are circled.

c | Flowchart of Cryo-EM micrographs processing for *Ce*CLHM1 and the FSC curves. Cyan and blue boxes in 2D class-averages show heterogeneous packing forms of CeCLHM1.

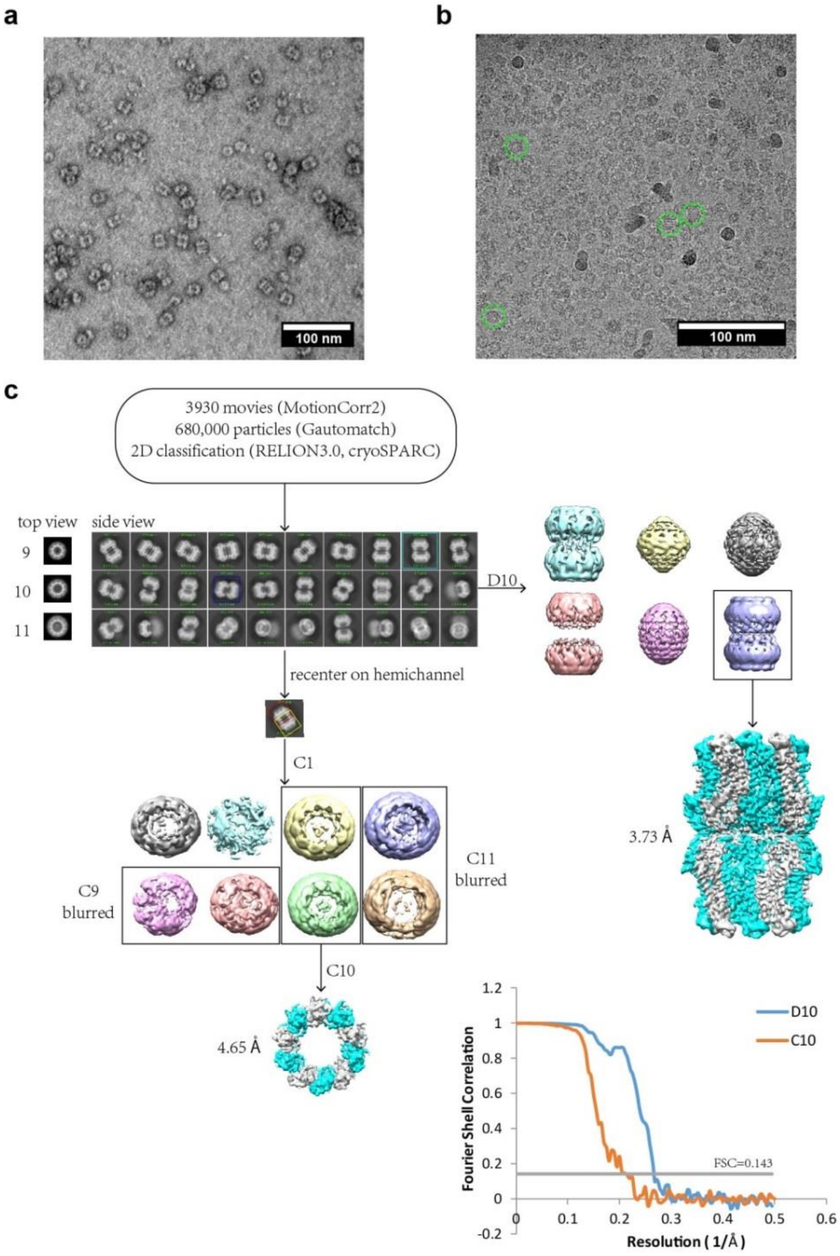

Figure S6. Representative densities and cryo-EM analysis of CeCLHM1 reconstituted in LMNG

a | Sample maps at three transmembrane helices (S0–S4) and intracellular helices (CH1, CH3, and CH4), contoured at 0.26 σ threshold level.

b | Local resolution estimation of 20-mer *Ce*CLHM1-BPY, calculated from Relion procedure.

c | The angular distribution of particles used for the refinement of *Ce*CLHM1-BPY.

d | S0-S1 packing in the inner layer of the channel. The S0 and S1 helices are colored in blue and cyan, respectively. The map was locally filtered, generating S0-S1 densities at about 7-Å resolution, and is contoured at 0.20 σ level.

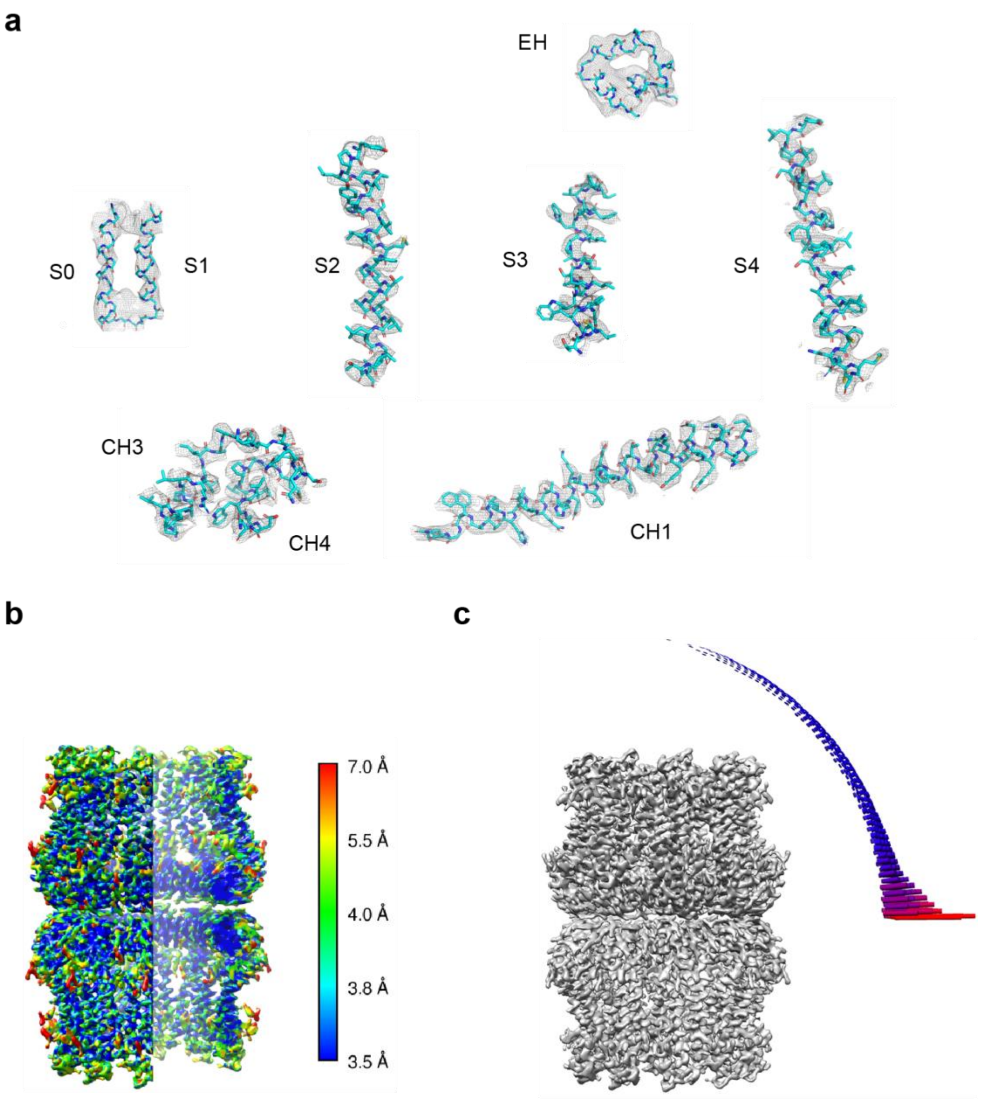

d.

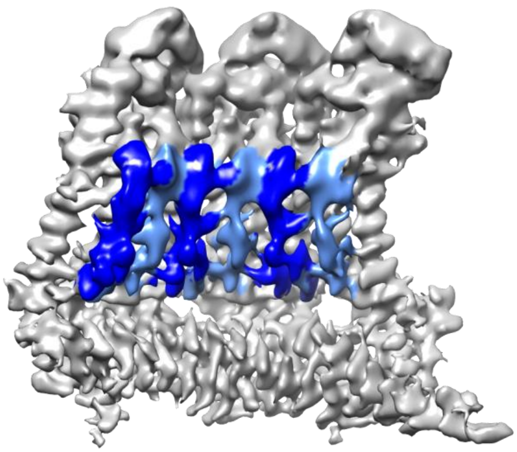

Figure S7. Charge distribution in the CeCLHM1 model.

a | The overall view of the compete model composed of 20-subunits with charge distribution.

b | The interior view of model docked by two *Ce*CLHM1 with charge distribution.

c | The exterior view of single layer model with charge distribution.

d | Charge distribution viewed from the CTD.

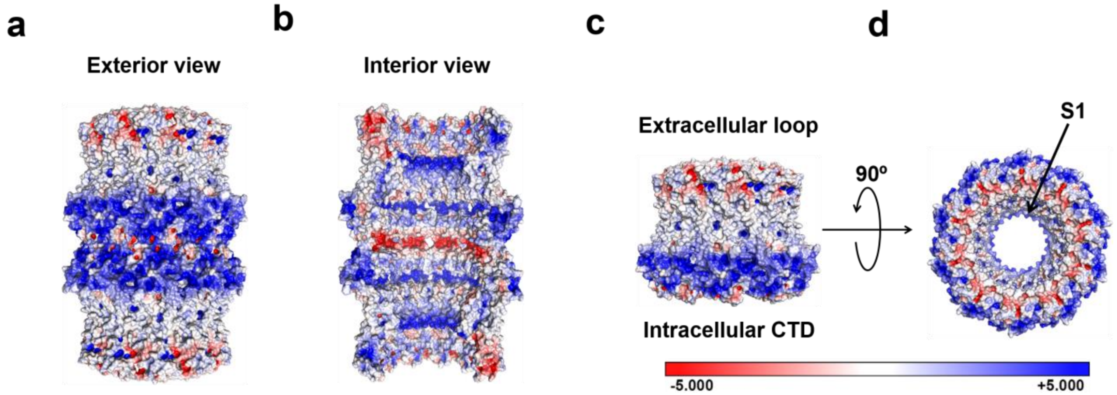

Figure S8. Location of CLHM1-GFP in the HEK293T cell and C. elegans.

a | Confocal microscopy analysis on CLHM1 expression in HEK293 cells. The upper group shows that CLHM1 is expressed in the plasma member. The lower group shows that, in a portion of cells, the CLHM1 is expressed in mitochondria.

b | Confocal microscopy analysis on CLHM1 expression in different tissues of *C. elegan*s. Green fluorescence indicates the *Ce*CLHM1 protein, and the red mAKAP1-mRFP specifically labels mitochondria.

a

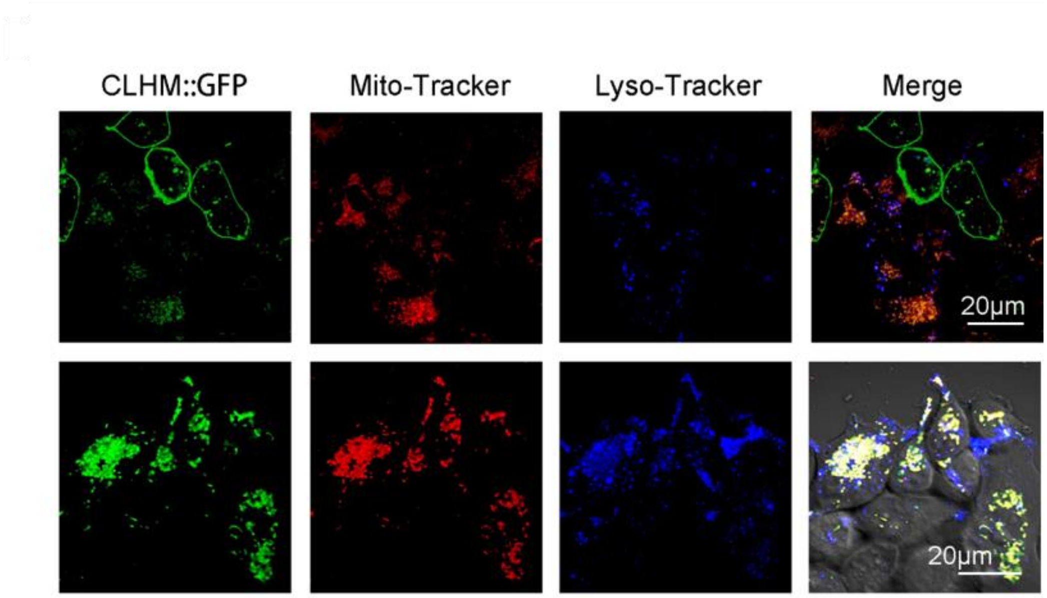
CLHM::GFP
Mito-Tracker
Lyso-Tracker
Merge
20μm
20μm

b

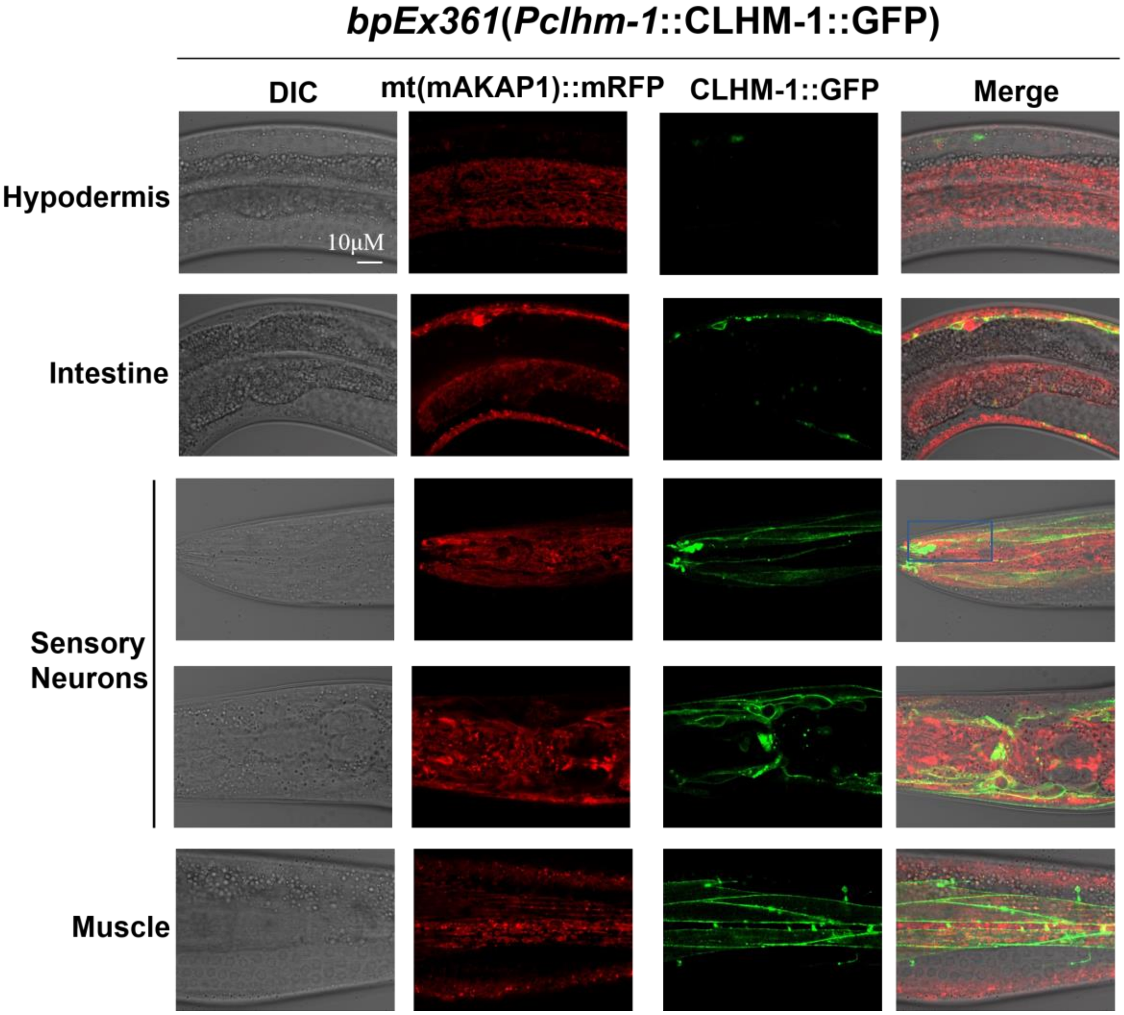
bpEx361(Pclhm-1::CLHM-1::GFP)
DIC
mt(mAKAP1)::mRFP
CLHM-1::GFP
Merge
Hypodermis
10μM
Intestine
Sensory Neurons
Muscle

Figure S9. Confocal images of three types of mutations

The CeCLHM1 variants are tagged with C-terminal GFP. The Alexa546 (labeled as A546) emits red light at 546 nm. Bar size is 30 µm.

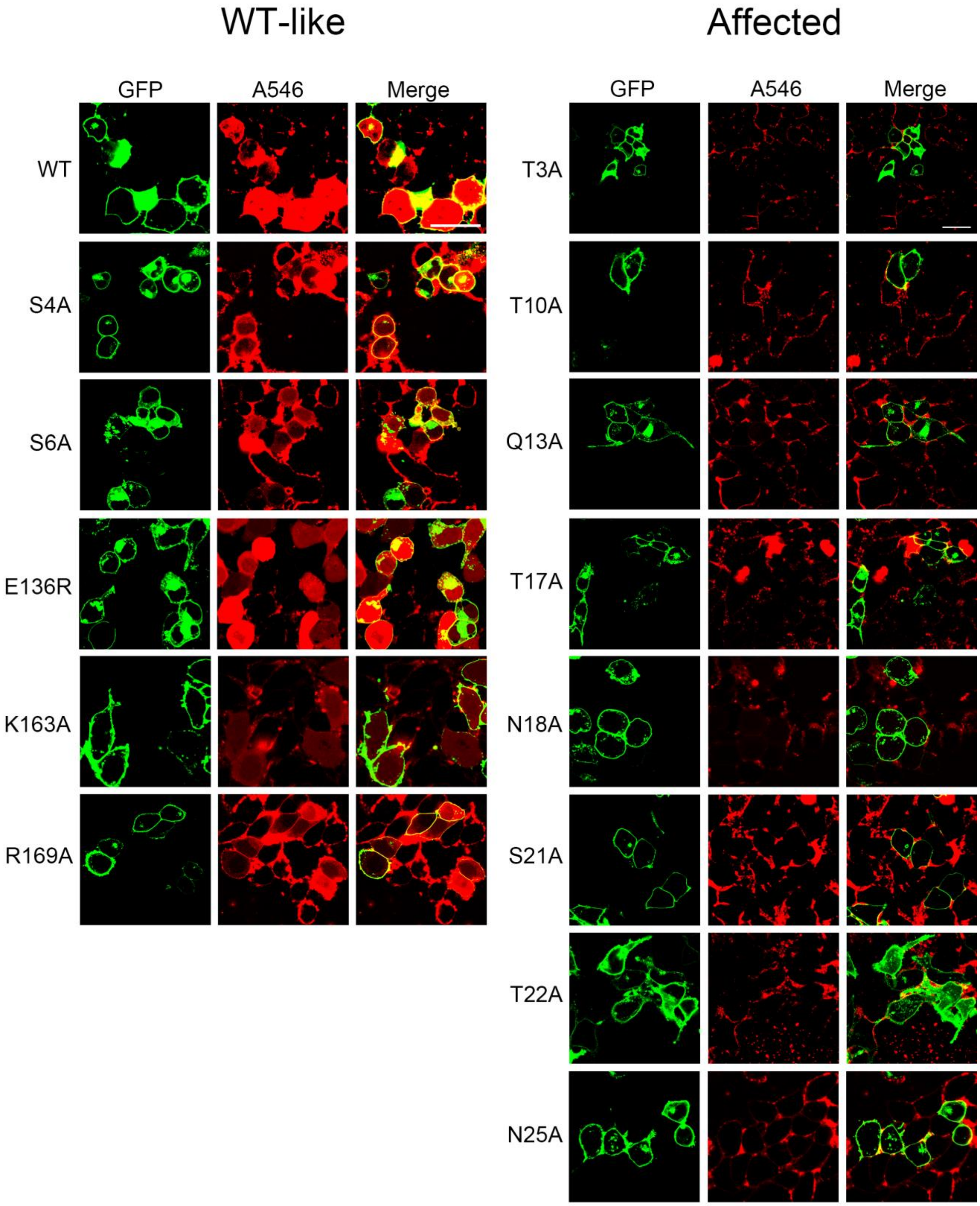

101 *(Fig. S9, 2/4)*

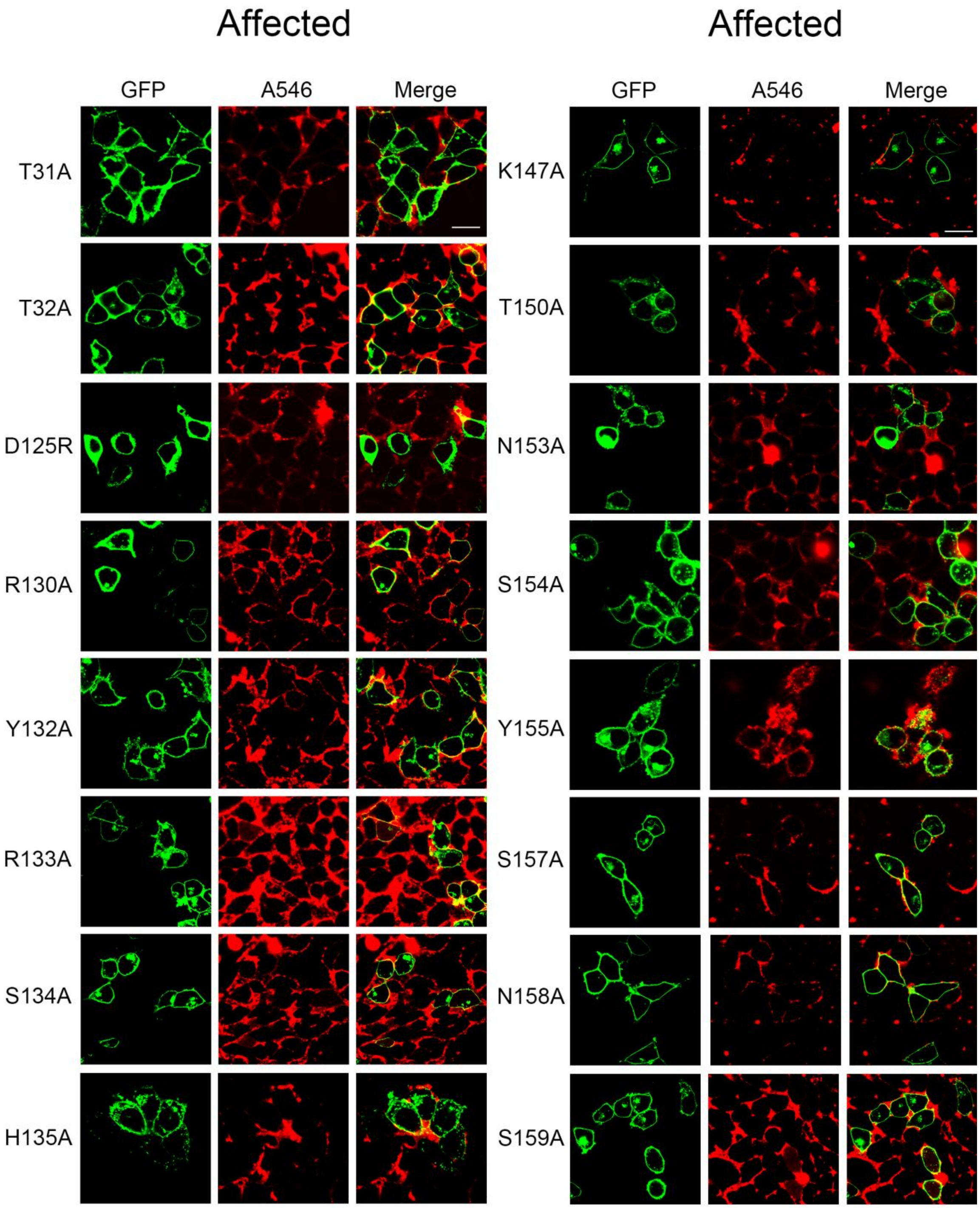

102

103 *(Fig. S9, 3/4)*

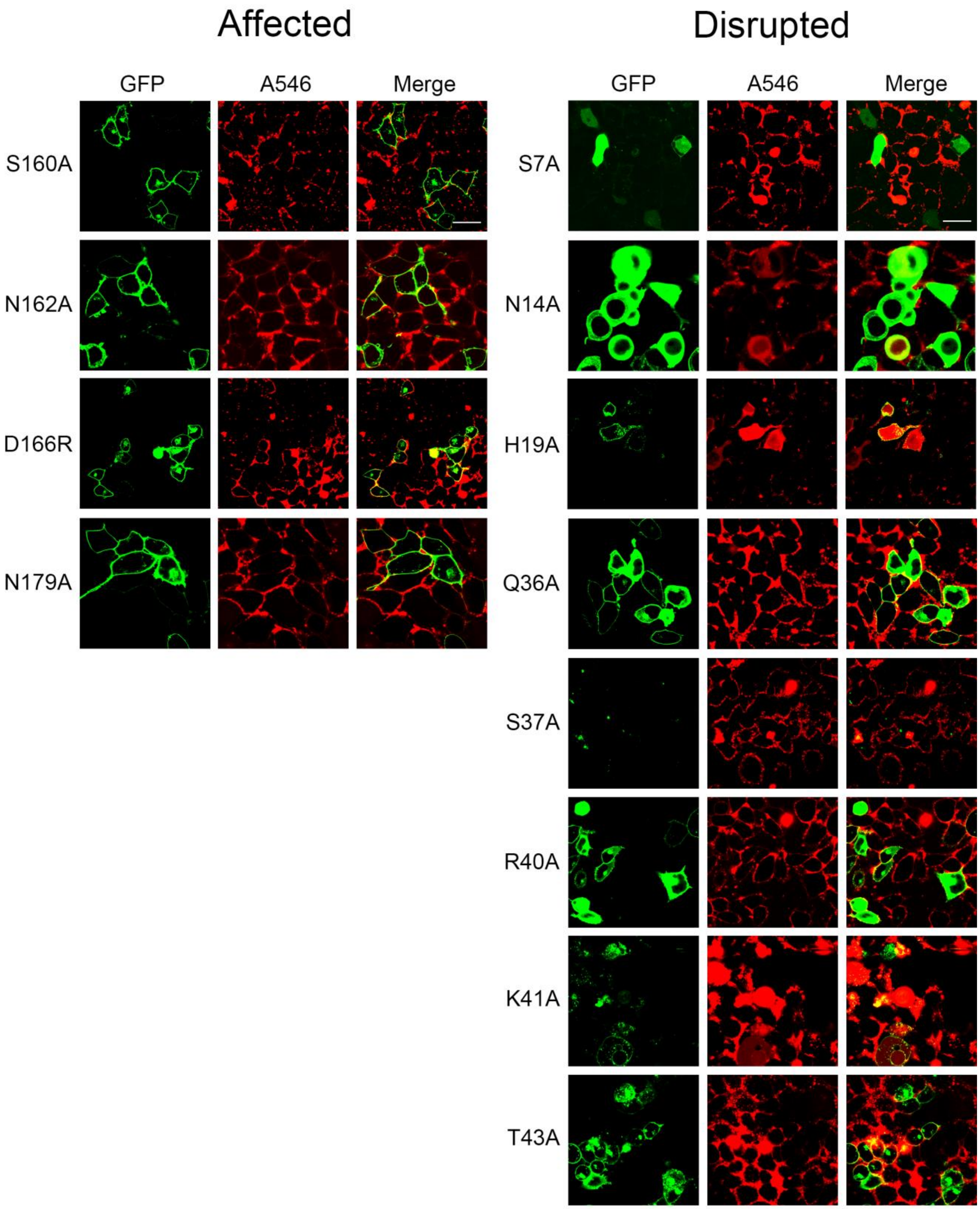

104

105 *(Fig. S9, 4/4)*

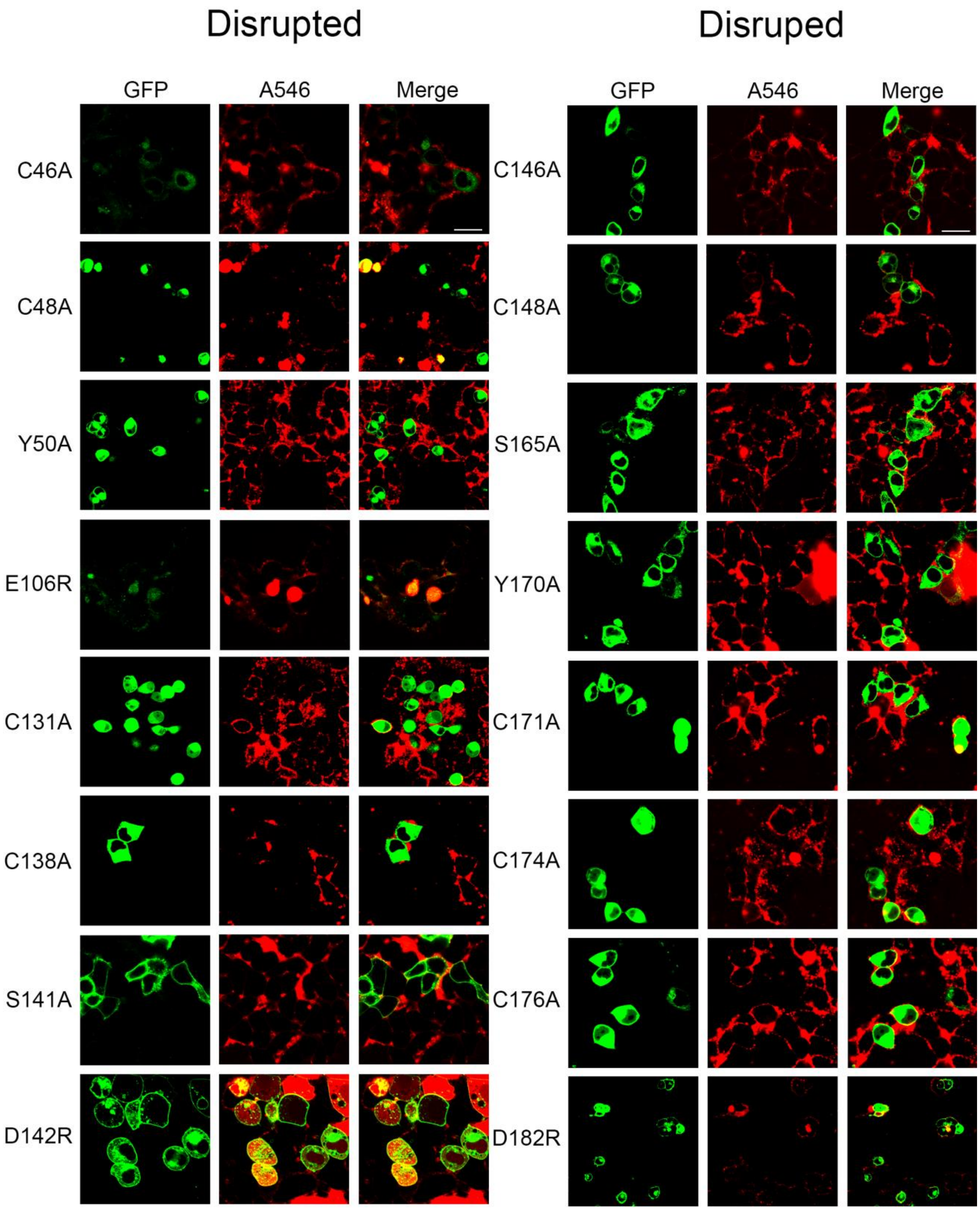

106

Figure S10. Comparison of CeCLHM1 with other CALHM channels

a | Overall structure comparison of *Ce*CLHM1 (6LOM; green) and hCALHM2-RUR (closed form; PDB ID: 6UIW; marine color), hCALHM2 (open; 6UIV; firebrick color), hCALHM6 (6YTV; magenta), and chicken CALHM1 (6VAM; orange). The upper group, side view; the lower group, top view, showing differences in size and pore diameter.

b | Comparison of two-layer structures *Ce*CALHM1, hCALHM2, and hCALHM4 (6YTK; cyan), showing differences in packing mode. *Ce*CLHM1 in green; hCALHM2 (6UIX) in marine color; right, hCALHM4 (6YTK) in cyan.

c | Superimpose of subunits of *Ce*CLHM1 and homologs, showing conformational change, including those in N-terminal domain (NTD, *i.e.* S0 and S1) and extracellular domain (ECD). The colors corresponding to proteins are the same as "*a"* and *"b"*.

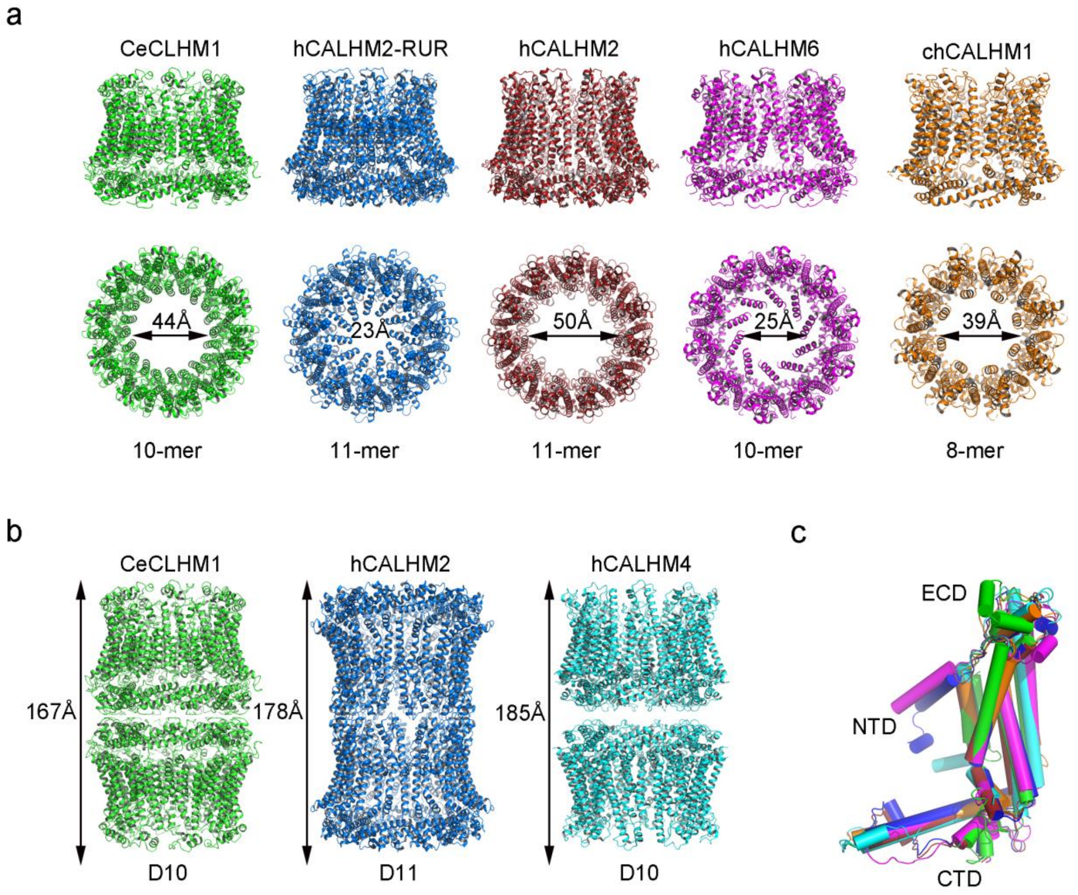

Figure S11. Structure comparison between CeCLHM1 and hCALHM4.

a | Superposition of 20-mers of *Ce*CLHM1 (gray) and hCALHM4 (blue). The gap between the two layers of hCALHM4 appears to be 10 Å wider than that of *Ce*CLHM1.

b | Superposition of the protomers of *Ce*CLHM1 and hCALHM4.

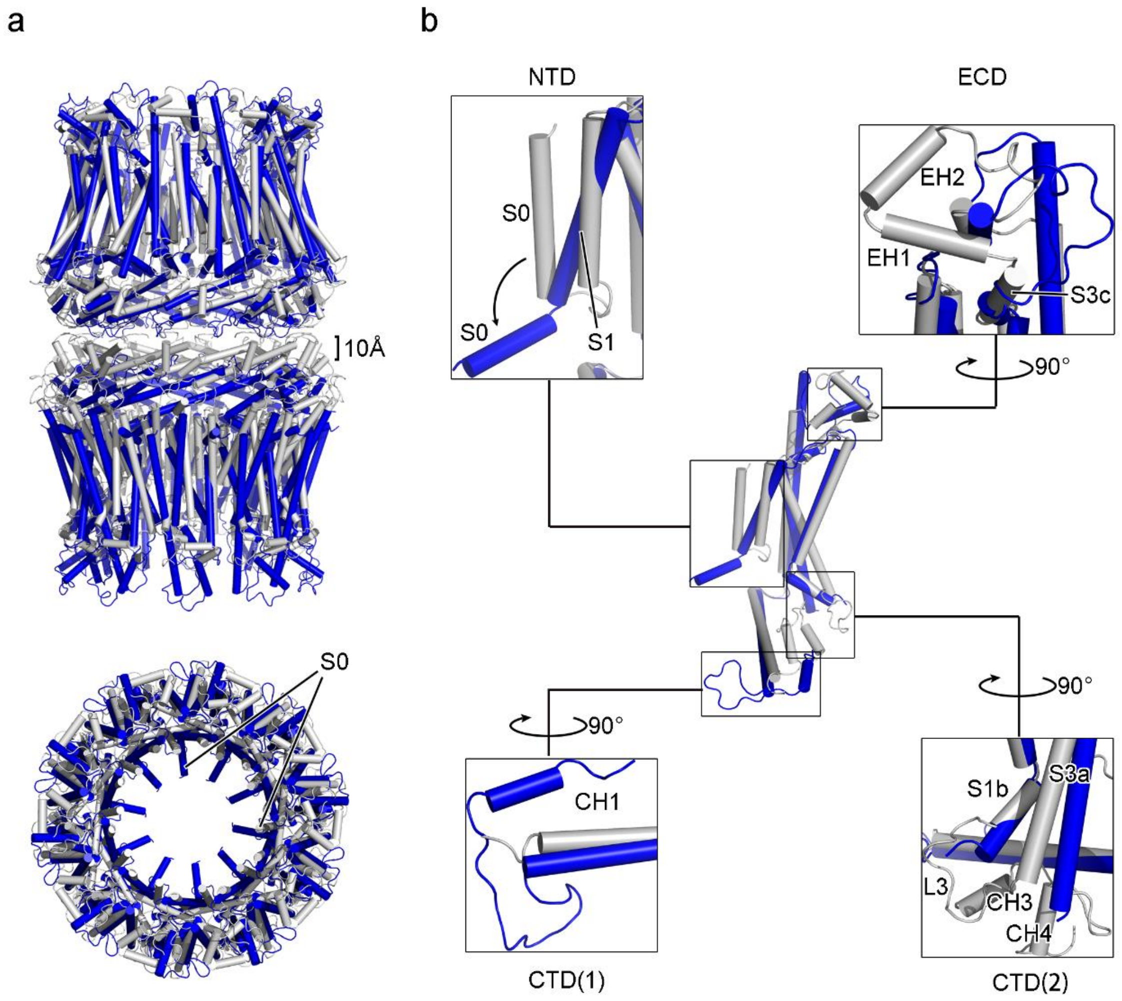

Supplementary Tables

Table S1. Summary of currently available CALHM/CLHM structures

Protein (source)	Resolution (Å)	PDB ID	Oligomerization	minimum diameter (Å)	detegent etc.	protomer r.m.s.d. (Å) / No. C_{α}-pairs	References
CALHM2 (human)	3.3	6UIV	undecamer	50	digitonin /EDTA	1.3 /192	Choi et al. *Nature* 2019
CALHM2 (human)	3.5	6UIX	undecamer x2, head-to-head	50	digitonin /EDTA	1.3 /180	Choi et al. *Nature* 2019
CALHM2 (human)	2.7	6UIW	undecamer	23	digitonin/BUR	1.3 /187	Choi et al. *Nature* 2019
CLHM1 (C. elegans)	3.6		nanomer	44	amphipol PMAL-C8		Demura et al. *BioRxiv* 2020
CALHM1 (killifish)	2.66		octamer	16	GDN		Demura et al. *BioRxiv* 2020
CALHM2 (human)	3.51		undecamer	64	digitonin		Demura et al. *BioRxiv* 2020
CALHM4 (human)	4.07	6YTK	decamer x2, tail-to-tail	25	GDN	1.5 /150	Drożdżyk et al. *Elife 2020*
CALHM4 (human)	3.82	6YTL	undecamer x2, tail-to-tail	30	GDN	1.4 /147	Drożdżyk et al. *Elife 2020*
CALHM6 (human)	4.39	6YTV	decamer	25	GDN	1.2 /180	Drożdżyk et al. *Elife 2020*
CALHM6 (human)	6.23	6YTX	undecamer	30	GDN	1.2 /180	Drożdżyk et al. *Elife 2020*
CALHM1 (chicken)	3.63	6VAM	octamer	39	nanodisc	1.6 /139	Syrjanen et al. *NSMB 2020*
CALHM2 (human)	3.48	6VAK	undecamer	61	nanodisc	1.4 /185	Syrjanen et al. *NSMB 2020*
CALHM2 (human)	3.68	6VAI	undecamer x2, head-to-head	60	nanodisc	1.4 /200	Syrjanen et al. *NSMB 2020*
CLHM1 (C. elegans)	3.73	6LOM	decamer x2, tail-to-tail (nanomer/undecamer)	44	LMNG /BPY		Yang et al. 2020

Table S2. Data collection and refinement statistics

Dataset	CalHM1-BPY
**Data collection**	
Microscope	FEI Talos Arctica
Voltage (kV)	200
Camera	Gatan K2 Summit
Magnification	22,500
Pixel size (Å)	1.00
Electron exposure (e^{-}/Å^{2})	60
Defocus range (μm)	-1.8 to -2.5
**Reconstruction**	
Software	*RELION 3.0*<br>*cryoSPARC*
Initial particle images (No.)	680,000
Final particle images (No.)	49,000
Symmetry imposed	D10
Final resolution (Å)	3.73
FSC threshold	0.143
Map-sharpening *B* factor (Å^{2})	-255
Map resolution range (Å)	3.5–7.0
**Model building**	
Software	*Coot*
**Refinement**	
Software	*Phenix*
Initial model used	de novo
CC_mask	0.76
Bond lengths (Å)	0.007
Bond angles (°)	1.108
**Validation**	
Clashscore	8.59
Rotamers outliers (%)	0.34
**Ramachandran plot**	
Favored (%)	91.41
Allowed (%)	8.25
Outliers (%)	0.34
**Detergent**	LMNG
**PDB code**	6LOM
**EMDB code**	EMD-0938

Table S3. Classification of 57 mutants

Types	Confocal images	Mutants
WT-like (5)	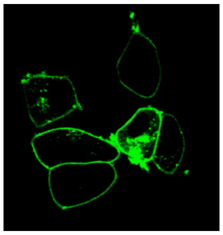	S4A, N6A, E136R, K163A, and R169A
Affected (28)	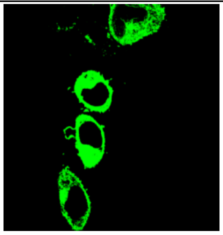 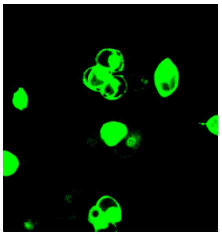	T3A, T10A, Q13A, T17A, S21, T22A, T32A, R130A, Y132A, R133A, S134A, H135A, K147A, T150A, N153A, S154A, Y155A, S157A, N158A, S159A, S160A, E166R, and N179A
	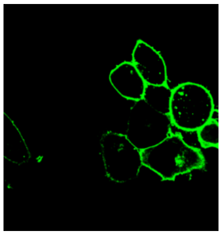	N18A, N25A, T31A, D125R, and N162A
Disrupted (24)	Several types	S7A, N14A, H19A, Q36A, S37A, R40A, K41A, T43A, C46A, C48A, Y50A, E106R, C131A, C138A, S141A, D142R, C146A, N148A, S165A, Y170A, C171A, C174A, C176A, and D182R

References

1. F. Madeira *et al.*, The EMBL-EBI search and sequence analysis tools APIs in 2019. *Nucleic acids research* **47**, W636-W641 (2019).
